# Identifying resting locations of a small elusive forest carnivore using a two-stage model accounting for GPS measurement error and hidden behavioral states

**DOI:** 10.1186/s40462-021-00256-8

**Published:** 2021-04-06

**Authors:** Dalton J. Hance, Katie M. Moriarty, Bruce A. Hollen, Russell W. Perry

**Affiliations:** 1 US Geological Survey, Western Fisheries Research Center, Columbia River Research Laboratory, Cook, WA 98605 USA; 2 National Council for Air and Stream Improvement, Inc., Corvallis, OR USA; 3 USDI Bureau of Land Management State Office, Portland, OR USA

## Abstract

**Background:**

Studies of animal movement using location data are often faced with two challenges. First, time series of animal locations are likely to arise from multiple behavioral states (e.g., directed movement, resting) that cannot be observed directly. Second, location data can be affected by measurement error, including failed location fixes. Simultaneously addressing both problems in a single statistical model is analytically and computationally challenging. To both separate behavioral states and account for measurement error, we used a two-stage modeling approach to identify resting locations of fishers (*Pekania pennanti*) based on GPS and accelerometer data.

**Methods:**

We developed a two-stage modelling approach to estimate when and where GPS-collared fishers were resting for 21 separate collar deployments on 9 individuals in southern Oregon. For each deployment, we first fit independent hidden Markov models (HMMs) to the time series of accelerometer-derived activity measurements and apparent step lengths to identify periods of movement and resting. Treating the state assignments as given, we next fit a set of linear Gaussian state space models (SSMs) to estimate the location of each resting event.

**Results:**

Parameter estimates were similar across collar deployments. The HMMs successfully identified periods of resting and movement with posterior state assignment probabilities greater than 0.95 for 97% of all observations. On average, fishers were in the resting state 63% of the time. Rest events averaged 5 h (4.3 SD) and occurred most often at night. The SSMs allowed us to estimate the 95% credible ellipses with a median area of 0.12 ha for 3772 unique rest events. We identified 1176 geographically distinct rest locations; 13% of locations were used on > 1 occasion and 5% were used by > 1 fisher. Females and males traveled an average of 6.7 (3.5 SD) and 7.7 (6.8 SD) km/day, respectively.

**Conclusions:**

We demonstrated that if auxiliary data are available (e.g., accelerometer data), a two-stage approach can successfully resolve both problems of latent behavioral states and GPS measurement error. Our relatively simple two-stage method is repeatable, computationally efficient, and yields directly interpretable estimates of resting site locations that can be used to guide conservation decisions.

**Supplementary Information:**

The online version contains supplementary material available at 10.1186/s40462-021-00256-8.

## Background

Innovations in animal biologging technology in recent years have opened significant new avenues of research into animal behavior and their landscape use. Lightweight biotelemetry devices containing Global Positioning System (GPS) or similar technologies have increasingly been adopted by researchers targeting a variety of terrestrial and aquatic species to collect time series of individual animal locations. These telemetry devices often contain multiple sensors (e.g., thermometer, accelerometer), allowing time series of location data to be paired to matching time series of physiological measurements or other auxiliary information [1]. Innovations in technology have been followed by advancements in statistical methods to analyze telemetry data, resulting in a broad array of multivariate random walk-type models that estimate state-dependent movement parameters from location time series data [2, 3]. However, there is no generally applicable movement model for animal location data because each analysis must be tailored both to the set of research questions under study and the particular structure of a given dataset (e.g. regular or irregular recording intervals or availability of auxiliary data) [4]. Given these issues, examples of using statistical movement models to directly inform management of animal populations are limited.

Analyses of time series data arising from animal telemetry studies such as this one are often complicated by two competing challenges arising from different kinds of unobservable information. The first is that any sufficiently long time series of animal locations (“track”) is likely to result from multiple behaviors [5]. These behaviors are seldom observed directly, but each is associated with different characteristic movement patterns. Hidden Markov models (HMMs) address this challenge by assuming that observed variables (e.g., step length, turning angle) arise from a mixture of distinct probability distributions associated with latent discrete behavioral states. Serial dependence is explicitly modeled by allowing animals to transition between states at each timestep where the state-dependent transition probability is a parameter to be estimated [6]. For example, Morales et al. [7] developed an HMM for elk (*Cervus canadensis*) where tracks were assigned to “exploratory” or “encamped” behavioral modes based on step lengths and turning angles between successive GPS positions. Because of HMMs’ flexibility and power, there are many recent examples of their application to positional data [8–10], and specific software tools have been developed for analysis of movement data with HMMs [11, 12]. HMMs can include or be fit entirely to non-positional data [13–15], and inclusion of auxiliary data with positional data can improve inference from HMMs [1]. However, when applied to positional data, HMMs are only appropriate for time series data recorded with minimal error because measurement error can severely affect model inference when the scale of measurement error is large compared to the scale of movement [16].

Observation error and missing observations comprise the second major challenge to analysis of location data because, in both cases, the true location of an animal is not directly observed. State space models (SSMs) are flexible time series frameworks that, in the context of animal movement, treat location as a latent “state” to accommodate missing observations and estimate observation error about the true location at each timestep [17]. The term SSM is used broadly in the literature and in some cases HMMs may be considered a special case of SSMs [18]. Here, we use the term in a manner similar to Patterson et al. [4] to refer to a model where the latent state is continuous. To avoid confusion of the term “state” as used in the SSM, we refer to discrete latent HMM states as “behavioral states” and continuous latent SSM states as “latent locations”. When process and observation errors are assumed to be Gaussian, the Kalman filter (KF) can be used to efficiently fit models to animal location data [17]. For example, Johnson et al. [19] fit a linear SSM using the KF to estimate the true position from irregularly observed harbor seal (*Phoca vitulina*) locations. However, while Johnson et al. [19] demonstrate that SSM can be used to estimate behavioral state specific movement parameters, the behavioral state must be known in advance.

Although robust methods exist to deal with these two challenges separately, addressing both simultaneously remains difficult. For example, the HMM of Morales et al. [7] assumed that measurement error was negligible in scale relative to the observed movement, and the SSM of Johnson et al. [19] assumed that behavioral states were sufficiently described by an observed covariate. Jonsen et al. [20] developed a model accounting for both behavior switching and measurement error by allowing multiple observations to be assigned to a true location, thus allowing for the replication necessary to provide an estimate of measurement error. McClintock et al. [1] used a similar approach but included independent information on measurement error. Additionally, while closed-form likelihoods can be calculated for HMMs and SSMs separately, no such expression exists for models that propose to combine features of both [21]. This necessitates use of computationally intensive approaches such as Gibbs sampling, which are not guaranteed to converge even after weeks of sampling. For these reasons, McClintock [22] proposed a two-stage approach to impute missing and imperfectly observed locations using a SSM followed by a HMM to identify latent behavioral states.

We were interested in using biotelemetry data to characterize resting and movement behavior and to identify resting sites of GPS-collared fishers (*Pekania pennanti)* in the Oregon and California Revested Lands (O&C Lands) managed by the Bureau of Land Management (BLM) in southern Oregon, USA. This is a unique region for western fisher research owing to checkerboard of public and private ownerships that causes a pronounced juxtaposition in land management practices. Fishers are a medium-sized member of the weasel family, typically associated with closed-canopy, older forests and are considered by land managers as a sensitive species when planning management actions. These territorial animals are thought to have at least one resting bout per day, often in structures such as snags or tree hollows [23]. The locations and associated vegetation of such resting locations have been described as representing “the best source of information that resources managers can use to maintain or improve habitat conditions for fishers [24].” While biotelemetry has been applied to this small forest-dwelling carnivore in recent years [25, 26], including examples of behavioral state switching models [27, 28], much remains unknown in terms of their basic habitat and space use such as duration, timing and location of resting, and information such as average distance moved per day. Understanding fisher movement and resting patterns is a first step to determining what types of forest management are conducive to the persistence of fisher populations. We deployed GPS collars on 9 individuals on 21 occasions with a goal of describing basic movement ecology to inform management of this carnivore. We were particularly interested in identifying the location of resting sites as this is thought to be a limiting factor for this species [23]. For each deployment, our data consisted of regular time series of GPS fix attempts and accelerometer-derived activity summaries. This species is often associated with areas of dense forest cover and resting in cavities formed in trees, snags, logs, or under the snow, which contributed to failure of a sizeable proportion of attempted GPS-fixes in each time series, resulting in missing location data for some timesteps. The remaining GPS fixes were known to be observed with error.

Our research question required us to account for both latent behavioral states (i.e., separating periods of resting from periods of movement) and telemetry error to obtain robust estimates of potential resting locations. To resolve both problems, we implemented a two-stage approach similar to that of McClintock [22], but in the opposite order. We chose to use the HMM first because our ultimate goal was an estimate of resting site location, which required us to first identify when a fisher was resting in order to model where it was resting. We first identified periods of resting and movement from time series of activity and partially observed step lengths using an HMM. Treating the resulting behavioral state estimates as known, we then fit an SSM to generate estimates of the true location of the animal at each timestep. Our results allowed estimation of valuable natural history and behavior information from an elusive, forest dependent, small carnivore. In particular, our goals were to use these methods to summarize three characteristics valuable to scientists and forest managers: (1) locations of putative resting locations that may be considered as focal spots for conservation; (2) characteristics of visitation and re-use by one or more resting fishers as a potential characteristic of presumed importance; and (3) resting durations and activity patterns (e.g., diurnal vs. nocturnal) to help guide field efficiencies or biases.

## Methods

### Study animal

Fishers are forest-dwelling meso-carnivores whose range in North America and the western states has substantially declined [29, 30]. Fishers are associated with older forest elements such as large live trees, snags, and logs (e.g., [31]) and are a cavity denning obligate, thus they seek structures with specific shelter-providing cavities for parturition and kit development [32]. Fishers rest in similar structures throughout the year [23, 33], and resting locations have been used to monitor habitat availability and trends [34]. Fishers were recently proposed for listing as Threatened under the Endangered Species Act (ESA) throughout the western United States [35], although this listing was determined to be not warranted for the northern California and southern Oregon distinct population segment [36]. Fishers are designated as a Sensitive Oregon Conservation Strategy Species [37]. At the time of study initiation, little was known about the fisher population and habits in the southern Oregon Cascades (but see [38]). These fishers differ from others in the region as they were reintroduced from Minnesota and British Columbia (1961–1981 [39]), and thus are larger than other fishers in Oregon and California (male average 4.5 kg (0.8 SD); female 2.6 kg (0.2 SD)) and our scope of inference is limited to this reintroduced population.

### Study area

Land ownership in our study area is a patchy mosaic of federal (BLM and United States Forest Service), state, and private (primarily commercially-owned timberlands) lands. Thus, our study provided a unique opportunity to monitor fisher behavior across a range of variable forest management practices and across a range of seasonal variation. Pacific fishers are often associated with mixed conifer oak woodlands and are active during all times of year although snow may alter their movement patterns [40]. We conducted our study between 2015 and 2018 and during all four seasons. Elevations in this mountainous region range from 1200 to 2500 m. Forest vegetation types are primarily mixed-conifer stands with predominant tree species that include white fir (*Abies concolor*), Douglas-fir (*Pseudotsuga menziesii*), red fir (*Abies magnifica*), ponderosa pine (*Pinus ponderosa*), sugar pine (*Pinus lambertiana*), lodgepole pine (*Pinus contorta*), and incense cedar (*Calocedrus decurrens*). Natural openings include perennial meadows and frozen lakes during winter. Average annual snowfall for the Howard Prairie Dam weather station located at 1396 m elevation within the study area is 349 cm and average annual precipitation is 82 cm, which is an unusually high amount of snow for fisher populations in the western United States. Winter mean annual snow depth for the study period was 37 cm at the Howard Prairie weather station. Snow persisted from November to May each year in much of the core study area. Mean annual precipitation was 77 cm for 2015 and 2016 (Natural Resources Conservation Service, SNOTEL data 2015–2016).

### Data description

We first identified areas inhabited by fishers using baited remote cameras at randomly selected locations within a systematic grid and with strategic placement (see [41] for methods). At locations with fisher detections, we trapped, anesthetized, and handled fishers using established protocols [42]. We fitted GPS collars (W500 Wildlink GPS Logger, 60 g, ATS Isanti, MN) to 9 individual fishers. Data used in this study were retrieved by recapturing the animal and downloading the archived data. We also retrieved some data remotely with UHF transmission to ensure some information was gained if an animal was not recaptured, but these remotely sampled data were not used in this study as all collars were successfully recovered. Each individual fisher was tracked for between one and four collar deployments resulting in 21 total deployments. Given an expected collar battery life ranging from approximately 30 to 120 days, we designed our study to obtain telemetry data representative of different seasons to ensure our scope of inference was not limited to a single season in case of seasonal differences. The number of days each collar was operational ranged from 23 to 170 (Table 1). We retain collar deployment within each model to assess the degree of variation between collars and individual behavior.

**Table 1:** Summary of 21 collar deployments for 9 fishers (*Pekania pennanti*) in the southern Oregon Cascades. Fisher ID is the unique identification assigned to each individual fisher with IDs beginning with F indicating females and beginning with M indicating males. Deployment indicates the season and year of the collar deployment. In 2016 there were two deployments during the summer, early summer deployments in 2016 (e.g beginning in late June) are indicated with the suffix “a” (Summer 2016a) and late summer deployments (e.g beginning in late August) are indicated with the suffix “b”. Number of fix attempts is the total length of the time series. Fix rate is the percent of the times series for which a GPS location fix was successful. Fix interval is the programmed interval at which GPS fixes were attempted. Average apparent step length (and standard deviation) were calculated based on observed step lengths (Euclidean distance) between successive successful fix attempts

ID	Deployment	Dates (day/month)	Duration (days)	Number of fix attempts	Fix rate	Fix interval (mins)	Average apparent step length [SD] (m)
F01T	Fall 2015	21/10–03/12	43	4092	0.79	15	73.5 (124.3)
F01T	Summer 2016a	27/06–05/08	39	3766	0.76	15	142.8 (141)
F01T	Summer 2016b	18/08–19/09	32	3028	0.64	15	112.1 (145.2)
F01T	Winter 2017	14/12–11/01	28	2650	0.71	15	108.1 (175)
F02T	Fall 2015	22/10–30/11	39	3728	0.80	15	66.5 (93.3)
F02T	Summer 2016b	17/08–26/09	40	3858	0.76	15	62.8 (97.3)
F03T	Fall 2015	25/10–30/11	36	3436	0.81	15	59.5 (110.8)
F03T	Summer 2016a	27/06–08/08	42	4036	0.80	15	141.3 (174.4)
F03T	Summer 2016b	23/08–15/09	23	2190	0.90	15	115 (149.8)
F03T	Winter 2017	07/11–03/12	26	2453	0.58	15	88.9 (153.5)
F07T	Fall 2016	09/11–04/12	25	2412	0.48	15	123.5 (161.8)
F07T	Fall 2017	20/08–23/09	35	1661	0.79	30	157.1 (229.5)
M03T	Fall 2016	10/11–01/02	83	7944	0.77	15	140.2 (221)
M03T	Winter 2017	17/02–31/05	103	4939	0.82	30	245.7 (456.5)
M05T	Fall 2016	23/10–17/11	25	2390	0.70	15	126.4 (166)
M07T	Fall 2016	18/10–22/11	35	3352	0.64	15	141.7 (174.3)
M08T	Fall 2016	13/11–18/12	35	3353	0.78	15	126.9 (212.4)
M08T	Spring 2018	12/02–30/07	168	8053	0.87	30	96.3 (242.4)
M08T	Winter 2017	08/02–26/06	137	6597	0.72	30	141.7 (330.3)
M09T	Spring 2018	01/03–25/06	116	5583	0.66	30	255.2 (478.6)
M09T	Winter 2017	10/02–14/03	32	1524	0.66	30	360.5 (593)

**Fig. 1:**
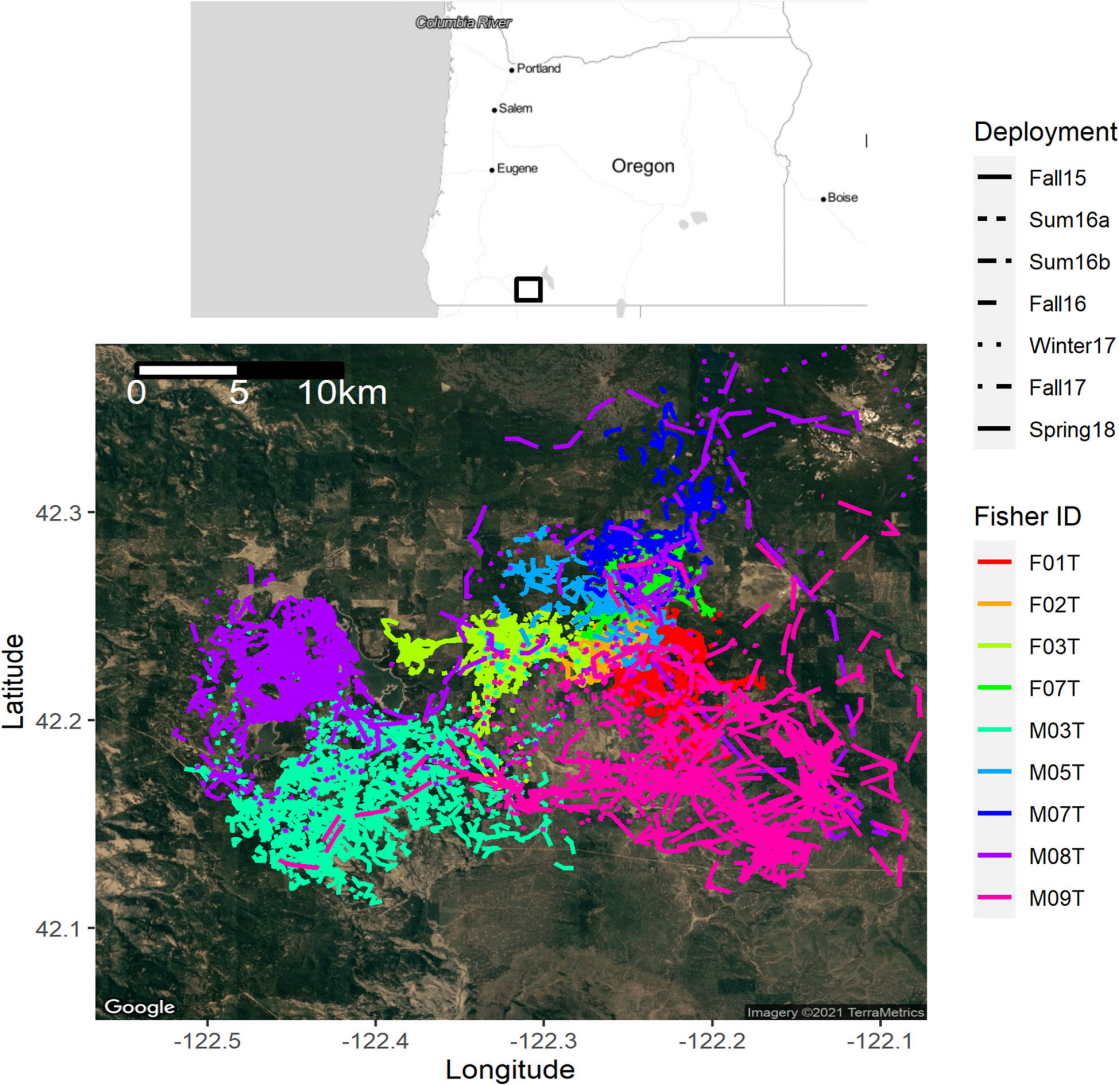
Observed GPS coordinates for 21 separate collar deployments on nine individual fishers in the study area of the Oregon and California Revested Lands in southern Oregon, USA

For each deployment, we programmed the collar to attempt a GPS location fix every 15 or 30 min, with the longer time interval generally corresponding to longer-duration deployments. If the collar was unable to obtain a GPS position fix after 180 s the collar was programmed to abort the fix attempt and no location was recorded. The fix success rate varied among collar deployments with between 10 and 55% of attempted position acquisitions failing. However, failed acquisitions more commonly occurred at the end of a deployment when battery power was diminishing. Thus, we truncated each time series by eliminating all observations after the last three consecutive successful location fixes. Fix rate in remaining times series ranged from 48 to 90% (Table 1). Missing location data were interspersed throughout each remaining time series. The average duration of data with consecutive successful GPS fixes before a failure was 6.75 h, but this varied among deployments (range of means: 2.75 to 21.5 h). Fishers are territorial carnivores and observed fisher GPS locations were generally confined to areas consistent with distinct territories with limited overlap between individuals. Territories of individual fishers were consistent across deployments (Fig. 1).

Each GPS collar contained a triaxial low-g accelerometer. The Wildlink collar is programmed to summarize accelerometer measurements by an “activity” variable. To create this variable, the accelerometer is sampled every second to detect a minimum acceleration of 0.77 ms^− 2^ in any direction and activity is summarized as the percent of seconds between each attempted GPS acquisition in which this minimum acceleration was detected. For example, for collars set to a 15-min interval, an activity measurement of 0.5 corresponded to 450 of 900 s for which the accelerometer recorded movement (personal communication, ATS Isanti, MN). Activity values ranged from 0.005 to 0.995 and were recorded for each time-step even if the GPS fix failed.

**Fig. 2:**
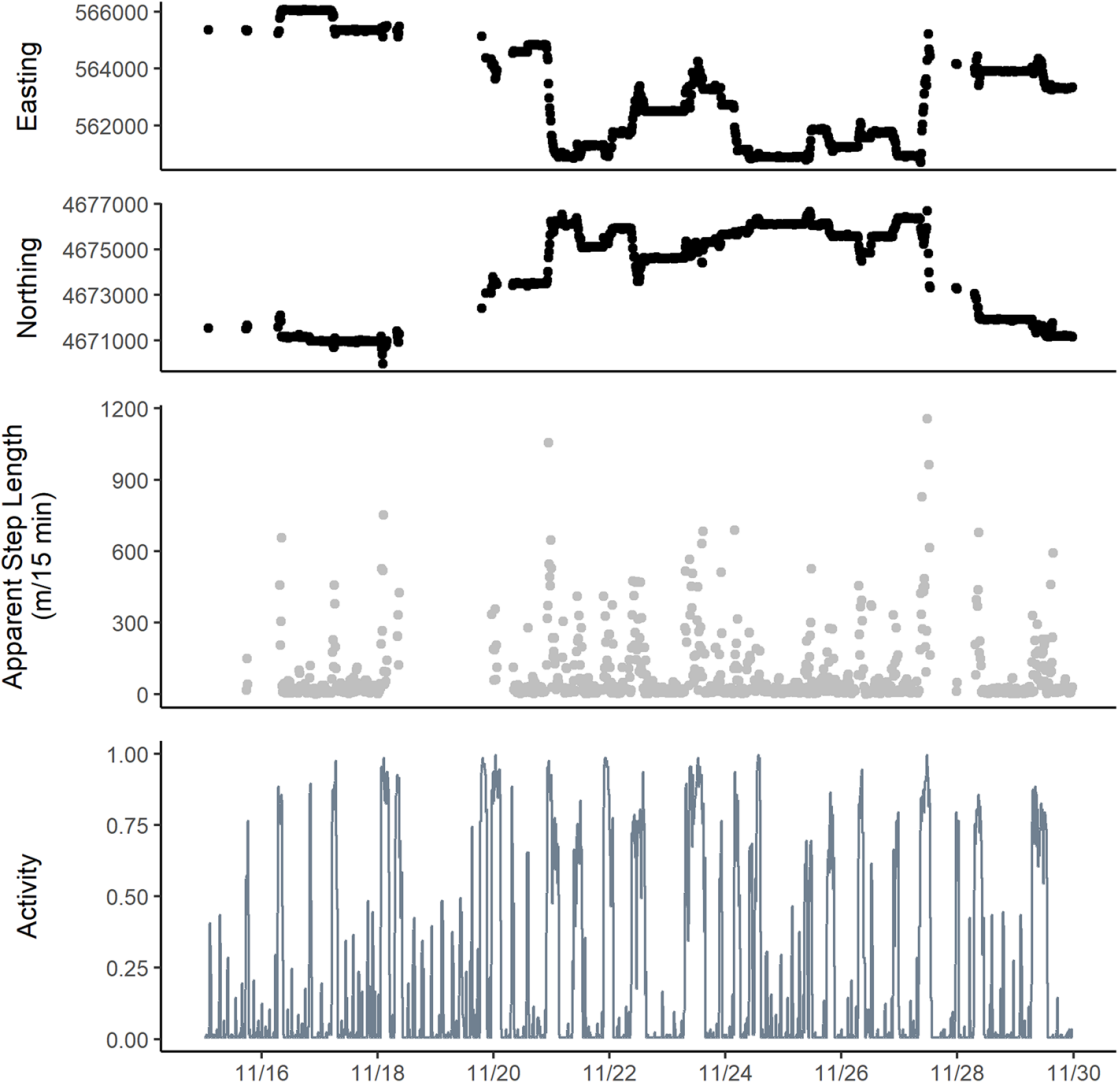
Excerpt of time series data for a single collar deployment (F01T Fall 15). Top two panels are observed Easting and Northings in meters. Gaps are due to missing data from failed GPS fixes. Third panel is apparent step length between successive GPS fixes. Bottom panel is accelerometer derived activity which summarizes the percent of time between successive GPS fix attempts the accelerometer detected movement exceeding a minimum acceleration threshold. Activity data are complete for each time series. This excerpt displays 14 of 42 days of the time series for this collar deployment

For each collar deployment, we had two related time series: the set of GPS fix attempts including missing GPS positions; and the set of activity measurements representing sequential pairings of consecutive GPS fix attempts. We use *t* as the time index for both time series with the understanding that data in the time-series representing summaries over the interval *t* to *t* + 1 map to *t*. For example, the activity measurement (*a*_*t*_) for time *t* = 1 represents the summary of accelerometer readings between *t* = 1 and *t* = 2. We define the vector ***x***_***t***_ ***=*** [*x*_1, *t*_, *x*_2, *t*_]^′^ as, respectively, the observed easting and northing GPS coordinates at time *t*. From this time series, we calculated apparent step lengths (*l*_*t*_) as stepwise Euclidean distance (e.g. ${l}_t=\sqrt[2]{{\left({x}_{1,t+1}-{x}_{1,t}\right)}^2+{\left({x}_{2,t+1}-{x}_{2,t}\right)}^2}$). We use the term “apparent step length” to indicate that the observed step lengths do not necessarily indicate actual movement but result in part from GPS measurement error. If at least one GPS location was missing, we recorded an *NA* for *l*_*t*_. This resulted in a paired set of bivariate time series for each collar deployment (Fig. 2); the first consisting of the observed easting and northings including missing locations, and the second consisting of a complete time series of activity measurement paired with the time series of observed and missing apparent step lengths.

### Hidden Markov models

In the first stage of the analysis, we fit HMMs to the time series of activity measurements and apparent step lengths of each collar deployment to identify periods of resting and periods of movement. Numerous resources cover application of HMMs to animal movement (e.g. [6]). Here, we briefly review the model structure assuming a time-homogenous process and adapting the notation of Leos-Barajas and Michelot [12]. For clarity, in this section and the next, we define the models generally for a single collar deployment. We fit the models independently to each collar deployment, yielding 21 sets of estimates for all parameters defined below.

In an HMM, the observed data vector at time *t*, ***y***_*t*_, depends on the underlying discrete behavioral state of the animal, *s*_*t*_. Each element of ***y***_*t*_ is conditionally independent and drawn from probability distributions that depend on the behavioral state. That is, P(***y***_*t*_|*s*_*t*_ = *i*)~*f*_*i*_ (***y***_*t*_), where *f*_*i*_ is the probability distribution function of ***y***_***t***_ when the animal is in behavioral state *i*. Note that *f*_*i*_ is a multivariate joint probability distribution if ***y***_***t***_ is multivariate. If one assumes the contemporaneous conditional independence of the elements of ***y***_***t***_, then *f*_*i*_ can be expressed as the product of univariate distributions. HMMs assume that *s*_*t*_ can take on a finite number of distinct states (*s*_*t*_ = 1, …, *N*) and that transitions between behavioral states over time occur via a first-order Markov chain. This means that the behavioral state at time *t* depends only on the behavioral state at time *t* – 1; that is, *P*(*s*_*t*_|*s*_*t* − 1_, …, *s*_1_) = *P*(*s*_*t*_|*s*_*t* − 1_)*.* The probability of transitioning from behavioral state *i* at time *t* – 1 to behavioral state *j* at time *t* is *γ*_*i*, *j*_ = *P*(*s*_*t*_ = *j*|*s*_*t* − 1_ = *i*), which is an element of the *N x N* transition probability matrix, **Γ.** To complete specification of the model requires the initial probability distribution, ***δ***, which for a time-homogenous process can be the stationary distribution defined such that ***δ = δ*Γ**. The marginal likelihood of the observed data can then be written as:

$$ \mathcal{L}=\boldsymbol{\delta} \mathbf{P}\left({\boldsymbol{y}}_{\mathbf{1}}\right)\boldsymbol{\Gamma} \mathbf{P}\left({\boldsymbol{y}}_{\mathbf{2}}\right)\dots \boldsymbol{\Gamma} \mathbf{P}\left({\boldsymbol{y}}_{\boldsymbol{T}}\right){\mathbf{1}}^{\prime } $$

where **P**(***y***_***t***_) **=** diag(*f*_1_(***y***_***t***_), …*f*_*N*_(***y***_***t***_)) and **1** is a length-N vector of 1’s.

In terrestrial applications, animal movement HMMs have typically been applied to animal location data by decomposing GPS coordinates and modelling the step lengths and turning angles between consecutive locations (e.g. [7]). We reasoned that fishers would be almost entirely stationary during times of resting but recognized that our location data were subject to GPS measurement error, resulting in non-zero apparent step lengths and spurious turning angles [43]. Because GPS measurement error affects turning angles to a greater degree than step lengths [44], we chose to use only the accelerometer-derived activity measurements and apparent step lengths. We expected that resting would be associated with lower activity measurements and shorter apparent step lengths. Because *a*_*t*_ was constrained between 0 and 1, we modeled activity as Beta distributed, where 0 < *μ*_*i*_ < 1 is the mean and *ϕ*_*i*_ > 0 is the precision of a Beta distribution given behavioral state *i* under the parameterization of Ferrari and Cribari-Neto [45]. We used a lognormal distribution for apparent step lengths, where *m*_*i*_ is the log mean and *σ*_*i*_ > 0 is the log standard deviation of a lognormal distribution given behavioral state *i*. Assuming independence between activity and step length within each behavioral state, the conditional joint likelihood when both variables are observed is: *f*_*i*_(***y***_*t*_ ***=*** {*a*_*t*_, *l*_*t*_}) = *f*_*i*_(*a*_*t*_)*f*_*i*_(*l*_*t*_) and when only activity measurements are observed is: *f*_*i*_(***y***_*t*_ ***=*** {*a*_*t*_, *NA*}) = *f*_*i*_(*a*_*t*_). When ***x***_***t***_ ***= x***_***t +*** **1*,***_*l*_*t*_ ***=*** 0, which is inadmissible under the log normal distribution. In these instances, we set *l*_*t*_ = 1 for the HMM model, which affected 193 of 57,788 observed *l*_*t*_ measurements. Because we were mainly interested in separating resting from movement, we fit a two-state model under the assumption that all other types of behaviors that involved movement would be captured by the remaining behavioral state. We used a single time-homogenous parameter, *ψ*_*i*_ as the probability of remaining in state *i* and let *γ*_*i*, *i*_ = *ψ*_*i*_ and *γ*_*i*, *j*_ = 1 − *ψ*_*i*_. We refer to *ψ*_*i*_ as the “state persistence probability.”

We identified resting periods by estimating the underlying behavioral state sequence given the complete observed data and the estimated parameters, ***Θ***. We used the forward-filtering, backward-sampling (FFBS) algorithm to globally decode the posterior marginal probability of the behavioral state sequence [46]. While the Viterbi algorithm has commonly been used to decode the most likely state sequence from HMMs [6], we chose to use the FFBS over the Viterbi because the Viterbi calculates the jointly most probable state sequence, whereas the FFBS calculates the posterior state assignment probability for each observation. We wanted to be conservative in assigning observations to the resting state, so we chose the FFBS because it gave a wider estimate of uncertainty of the true state for each timestep. We assumed that the behavioral state associated with lower activity corresponded to resting, which we labeled: *s*_*t*_ = 1. For each collar deployment, we used the posterior distribution of decoded behavioral state sequences to assign the inferred behavioral state, ${s}_t^{\ast }=0\ \mathrm{if}\ P\left({s}_t=1\ |\ {\boldsymbol{y}}_{1,}\dots, {\boldsymbol{y}}_T,\boldsymbol{\varTheta} \right)>0.95;\mathrm{and}\ 1\ \mathrm{otherwise}.$

### State space models

Assuming the inferred behavioral states as given, we used a linear Gaussian SSM to estimate fisher resting locations and movement between resting locations while accounting for GPS measurement error. The linear Gaussian SSM assumes two conditional relationships: first, that the time series of observed locations, ***x***_***t***_ depends on a sequence of unobserved continuous latent locations ***α***_***t***_; and second, that ***α***_***t +*** **1**_ depends on ***α***_***t***_**.** Like HMMs, SSMs have been widely applied to animal movement data (e.g. [19]) to take advantage of the KF [17] to efficiently estimate parameters related to movement and measurement error and to estimate (“smooth”) the time series of latent locations from imperfectly observed coordinate data. Here, we briefly review the mathematical details as they pertain to our implementation.

The linear Gaussian SSM consists of two related equations, the observation equation and the system equation:

$$ {\boldsymbol{x}}_{\boldsymbol{t}}={\boldsymbol{Z}}_{\boldsymbol{t}}{\boldsymbol{\alpha}}_{\boldsymbol{t}}+{\boldsymbol{\varepsilon}}_{\boldsymbol{t}}\kern2.25em {\boldsymbol{\alpha}}_{\boldsymbol{t}+\mathbf{1}}=\kern0.5em {\boldsymbol{U}}_{\boldsymbol{t}}{\boldsymbol{\alpha}}_{\boldsymbol{t}}+{\boldsymbol{R}}_{\boldsymbol{t}}{\boldsymbol{\eta}}_{\boldsymbol{t}} $$

where ***x***_***t***_ are the observation locations, ***α***_***t***_ are the latent locations, ***Z***_***t***_ is the design matrix that maps ***α***_***t***_ to ***x***_***t***_, ***ε***_***t***_ is the mean-zero normally-distributed observation error vector with covariance matrix ***H***_***t***_, ***U***_***t***_ is the transition matrix mapping ***α***_***t***_ to ***α***_***t +*** **1**_, ***η***_***t***_ is the mean-zero normally-distributed process error vector with covariance matrix ***Q***_***t***_**,** and ***R***_***t***_ is the system selection matrix.

We parameterized our SSMs to alternate between periods of movement and resting based on the inferred behavioral state (${s}_t^{\ast }$) arising from the HMM. We assumed that the fisher was stationary during periods of resting (${s}_t^{\ast }=0$), meaning that ***α***_***t +*** **1**_ ***= α***_***t***_. During periods of movement (${s}_t^{\ast }=1$), we assumed that fisher moved according to a first-difference correlated random walk (DCRW) [21]. To account for the DCRW we set ***α***_***t***_ = [*α*_1, *t*_, *α*_2, *t*_, *α*_1, *t* − 1_, *α*_2, *t* − 1_]^′^ where *α*_1, *t*_ and *α*_2, *t*_ are, respectively, the latent easting and northing coordinates of the fisher at time *t*. For all *t*, we set the design matrix $$ {\boldsymbol{Z}}_{\boldsymbol{t}}=\left[\begin{array}{cccc}1& 0& 0& 0\\ {}0& 1& 0& 0\end{array}\right] $$ to reduce the four-element ***α***_***t***_ vector to the two-element ***x***_***t***_**.** We assumed a constant GPS error over time and independent in each direction, that is ***H***_***t***_ **=** $$ \left[\begin{array}{cc}h& 0\\ {}0& h\end{array}\right] $$ where *h* is the observation error standard deviation. Together, the transition matrix and system selection matrices encoded either non-movement or a DCRW with correlation coefficient, ρ, as:

$$ {\boldsymbol{U}}_{\boldsymbol{t}}=\left[\begin{array}{cccc}1+\uprho {s}_t^{\ast }& 0& -\uprho {s}_t^{\ast }& 0\\ {}0& 1+\uprho {s}_t^{\ast }& 0& -\uprho {s}_t^{\ast}\\ {}1& 0& 0& 0\\ {}0& 1& 0& 0\end{array}\right]\kern5.25em {\boldsymbol{R}}_{\boldsymbol{t}}=\left[\begin{array}{cc}{s}_t^{\ast }& 0\\ {}0& {s}_t^{\ast}\\ {}0& 0\\ {}0& 0\end{array}\right] $$

Finally, we assumed a two-element system disturbance vector, ***η***_***t***_ ***=*** [*η*_1, *t*_, *η*_2, *t*_]**,** representing movement in the easting and northing directions, respectively, with covariance matrix ***Q***_***t***_ **=** $$ \left[\begin{array}{cc}q& 0\\ {}0& q\end{array}\right] $$ where *q* is the process error (movement distance) standard deviation. Thus, when the fisher is inferred to be moving (${s}_t^{\ast }=1$) and using ***α***_***t***_ = [*α*_1, *t*_, *α*_2, *t*_, *α*_1, *t* − 1_, *α*_2, *t* − 1_]^′^ as the frame of reference, these relationships yield:

$$ {\boldsymbol{\alpha}}_{\boldsymbol{t}+\mathbf{1}}=\left[\begin{array}{c}{\alpha}_{1,t}+\uprho \left({\alpha}_{1,t}-{\alpha}_{1,t-1}\right)+{\eta}_{1,t}\\ {}{\alpha}_{2,t}+\uprho \left({\alpha}_{2,t}-{\alpha}_{2,t-1}\right)+{\eta}_{2,t}\\ {}{\alpha}_{1,t}\\ {}{\alpha}_{2,t}\end{array}\right] $$

which states that fisher location at time *t* + 1 is equal to the location at time *t* plus some contribution of its velocity from the previous timestep, as represented by the first-difference, *α*_1, *t*_ − *α*_1, *t* − 1_, plus some additional movement deviating from the velocity and direction of the last time step. When we inferred the fisher to be resting (${s}_t^{\ast }=0$), we have simply ***α***_***t +*** **1**_ = [*α*_1, *t*_, *α*_2, *t*_, *α*_1, *t*_, *α*_2, *t*_]^′^, which states that the fisher did not move in the previous timestep.

### Model fitting and inference

We fit both models in the Stan probabilistic programming language [47]. While several standalone R packages have been developed for animal movement models (e.g., [12]) and Bayesian models have been published using BUGS-type language (e.g., [20]), we chose to use Stan because of its speed and customizability [48]. We completed all data formatting and model fitting in R version 4.0.1 [49] using the rstan package [50]. We adapted the Stan code of Leos-Barajas and Michelot [13] for the HMMs and that of Arnold [51] for the SSMs.

We first fit independent HMM models to the time series of activity measurements and apparent step lengths for each collar deployment. We used identical semi-informative priors for each time series: logit(*μ*_1_)~*N*(−3, 0.5); logit(*μ*_2_)~*N*(0, 1); log(*ϕ*_1_)~*N*(1, 1); log(*ϕ*_2_)~*N*(0, 1); *m*_1_~*N*(3, 1); *m*_2_~*N*(5, 1); *σ*_1_~ ∣ *N*(3, 1) |; *σ*_2_~ ∣ *N*(3, 1) |; *ψ*_1_~*Unif*(0, 1); *ψ*_2_~*Unif*(0, 1). For each model, we ran four Hamiltonian Monte Carlo (HMC) chains with a warmup of 1000 iterations and sampled for 1000 iterations. We used semi-informative priors with the intention to avoid identifiability issues common to mixture models [52]. We selected priors based on preliminary graphical analysis of apparent step lengths and activity measurements. While our data structure of multiple animals with one or more collar deployments lends itself to a hierarchical model wherein the movement parameters for each time series are drawn from a common distribution, we chose to fit the model independently because our main focus for inference was on the location of each rest event rather than on variation of higher-level parameters across animals. Given the size of our data, we did not expect a hierarchical model to improve state assignment estimates [53].

Because general behavioral information on fishers is limited, we were interested in describing diurnal patterns of resting. We used the R package suncalc to compute the positions of the sun for our study area on days when GPS collars were deployed [54]. We defined eight categories of daylight: (1) “night after solar nadir” from the solar nadir to the beginning of astronomical twilight; (2) “morning twilight” from the beginning of astronomical twilight to dawn (the start of civil twilight); (3) “morning” from dawn to the end of the golden hour (approximately 1 h after the end of sunrise); (4) “day before solar noon” from the end of morning to solar noon; (5) “day after solar noon” from solar noon to the start of the evening golden hour; (6) “evening” from the end of the day through dusk (evening civil twilight); (7) “evening twilight” from dusk to the end of astronomical twilight; and (8) “night before solar nadir” from the end of twilight to the solar nadir. For each category, we calculated the proportion of each HMM time series classified as resting out of all time series observations.

We next fit independent SSM models to time series of GPS fix attempts and the inferred state assignment from the HMM. For the SSM models, we used identical weakly-informative priors that were uniform over the support of the parameters. We again ran four HMC chains with a warmup of 1000 iterations and sampled for 1000 iterations for each model. After fitting the SSM, we estimated the true location at each timestep. We used the mean-correction simulation smoother to draw samples from the posterior of true locations [17]. For each rest event, defined as a consecutive set of timestamps for which ${s}_t^{\ast }=0$, we used the ks package in R to estimate the 50, 80, and 95% credible ellipses [55] for the location associated with each rest event. We were also interested in identifying potential reuse of rest sites by one or multiple fishers because reuse of landscape features as resting sites or use by multiple fishers has not been well-documented and such features may be of greater conservation value than resting sites that are used only once. Out of all rest events identified across the 21 deployments, we selected those with at least one successful GPS fix, because preliminary analysis of the results showed that rest events with no successful GPS fixes had very large credible ellipses. We identified spatially unique rest locations by spatially joining overlapping 95% credible ellipses. We defined reuse of a rest location if rest events for two different fishers or rest events of the same fisher were separated by at least 24 h and had overlapping 95% credible ellipses.

We conducted a partial field validation of our method using the integrated VHF transmitter on the GPS collars which we used to opportunistically locate rest and den locations during field data collection. We acoustically determined whether collared fishers were inactive as indicated by a consistent VHF signal heard through a passive telemetry receiver (R-1000, Communication Specialists, Orange, California, USA). If the fisher was inactive for > 2 min, we attempted to locate the resting or denning fisher by “walking in” and decreasing the gain, or power, on the receiver as we approached the structure. The field observer rated their confidence of the location between 1 and 5 (1 = fisher visually seen in structure, 2 = signal consistent with one structure, 3 = fisher within 2–3 adjacent structures, 4 = visual of animal leaving but structure not identified, 5 = within 100 m of animal but structure not identified). We compared the VHF identified rest sites to our model output as a partial validation of our approach. For this comparison, we restricted our sample to VHF-locations with a confidence of 3 or less.

## Results

### Hidden Markov models

**Fig. 3:**
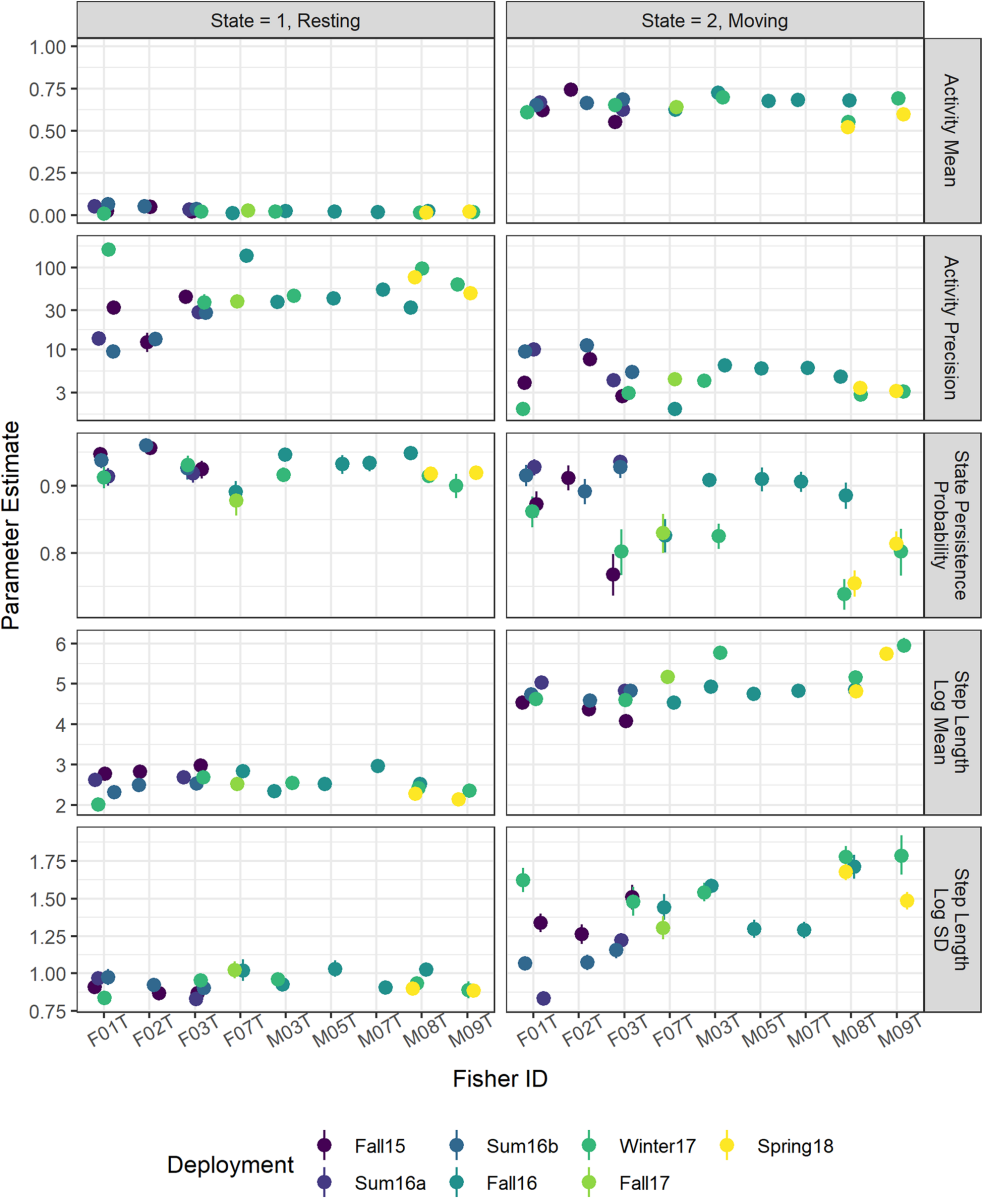
Parameter estimates from hidden Markov models (HMMs) fit to activity and apparent step lengths of 21 collar deployments for fishers. Left panel are estimates for when the fisher is in resting state and right panel is for the moving state. Parameters describe from top to bottom: log precision of the beta distribution for the activity measurement; logit mean of the beta distribution for the activity measurement; state maintenance probability is the probability of remaining in the same state in the next time step; log mean of the lognormal distribution for apparent step lengths and log standard deviation of the lognormal distribution for apparent step lengths. All estimates display the posterior mean with error bars representing the 90% uncertainty intervals

The HMM models fit independently to 21 collar deployments converged successfully to similar estimates that clearly discriminated between two latent behavioral states. The number of effective posterior samples for the main parameters of the models ranged from 777 to 7126 (mean 4142) and each parameter had $\hat{R}<1.01$, indicating successful convergence. Although the models were fit independently to each collar deployment time series, parameter estimates were qualitatively similar across all 21 models (Fig. 3), which suggests that the models describe a consistent suite of behaviors across the population of 9 fishers. Similarly for each collar deployment, the HMM models showed strong separation in the posterior probability of being in the presumed resting (*s*_*t*_ = 1) or moving states (*s*_*t*_ = 2), as only 3% of all observations had 0.05< *P*(*s*_*t*_ = 1) < 0.95 (range 1 to 7%; Table 2).

**Table 2:** Summary of resting state assignment using a two-state hidden Markov model fit to activity and apparent step-length data for 21 GPS collar deployments on 9 individual fishers. Percent of time resting is the percentage of time series for which P(*s*_*t*_ = 1) > 0.95. Percent uncertain is the percentage of time series for which P(*s*_*t*_ = 1) < 0.95 and P(*s*_*t*_ = 2) < 0.95. The number of rest events is the number of segments of the time series with sequential resting state assignments. The average duration (and standard deviation) of these rest events is indicated

Fisher ID	Deployment	Percent of time resting	Percent uncertain	Number of rest events	Average duration (SD) in hours
F01T	Fall 15	0.69	0.04	179	3.9 (3.0)
F01T	Summer 16a	0.44	0.03	145	2.9 (2.5)
F01T	Summer 16b	0.55	0.04	117	3.6 (3.1)
F01T	Winter 17	0.59	0.04	150	2.6 (2.1)
F02T	Fall 15	0.65	0.04	131	4.6 (3.9)
F02T	Summer 16b	0.72	0.02	132	5.2 (3.8)
F03T	Fall 15	0.72	0.07	228	2.7 (2.2)
F03T	Summer 16a	0.42	0.04	132	3.2 (3.0)
F03T	Summer 16b	0.49	0.02	78	3.4 (3.1)
F03T	Winter 17	0.72	0.05	144	3.0 (2.7)
F07T	Fall 16	0.59	0.04	165	2.2 (1.7)
F07T	Fall 17	0.57	0.02	119	4.0 (3.3)
M03T	Fall 16	0.62	0.02	286	4.3 (3.9)
M03T	Winter 17	0.67	0.02	295	5.6 (3.9)
M05T	Fall 16	0.56	0.02	95	3.5 (2.6)
M07T	Fall 16	0.58	0.01	134	3.6 (3.3)
M08T	Fall 16	0.67	0.03	127	4.4 (4.6)
M08T	Spring 18	0.73	0.04	533	5.5 (4.4)
M08T	Winter 17	0.73	0.03	442	5.5 (5.0)
M09T	Spring 18	0.68	0.03	335	5.7 (4.5)
M09T	Winter 17	0.65	0.03	101	4.9 (3.3)

**Fig. 4:**
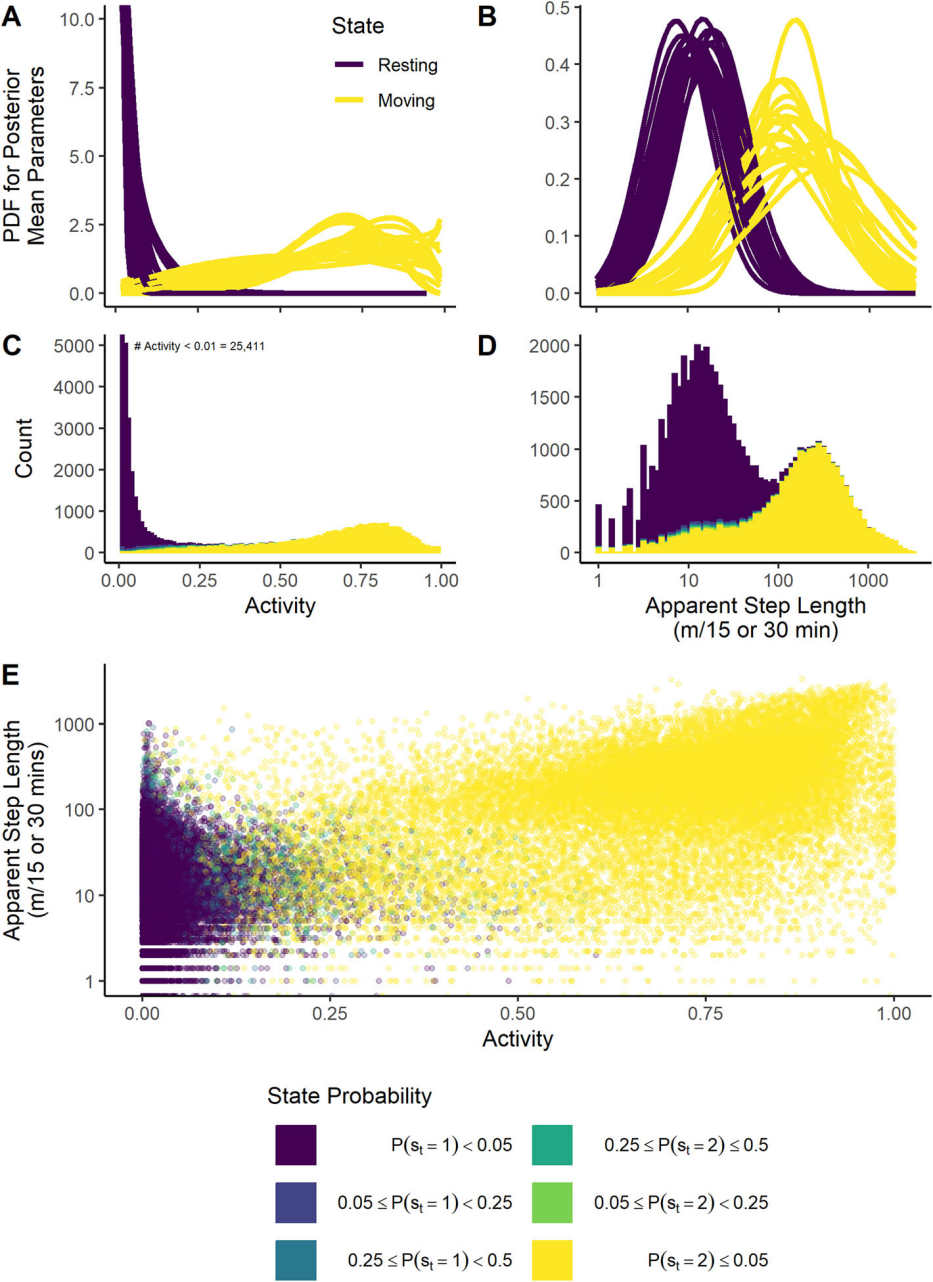
Posterior mean probability distribution functions (PDF) and observed histograms of activity and apparent step lengths colored by posterior state assignment probability. The top panel depicts the PDFs of the activity (left) and apparent step lengths (right) evaluated at the posterior mean for parameter for each of 21 collar deployments. Apparent step lengths are modeled as lognormal and plotted on a log_10_ axis. Middle panels display aggregated histograms across all 21 collars deployments for activity (left) and apparent step lengths (right) colored in bins by posterior state assignment probability. Note: for the activity measurement 25,411 of 81,024 time series observations had an observed activity measurement of 0.005 which is beyond the scale of the y-axis. Bottom panel displays a scatter plot of apparent step lengths versus activity color by posterior state assignment probability

The resting state was strongly associated with small values of *a*_*t*_. Across 21 collar deployments, the posterior mean of the Beta distribution mean for the resting state (*μ*_1_) ranged from 0.01 to 0.06 (average 0.03) and precision (*ϕ*_1_) ranged from 9.5 to 164 (average 50). These parameters resulted in a set of Beta distributions for the resting state with a mode near 0 and with little probability density for activity values greater than 0.25 (Fig. 4). For the moving state, the posterior mean of *μ*_2_ ranged from 0.52 to 0.75 (average 0.65). However, precisions (*ϕ*_2_) were less for the moving state, ranging from 1.9 to 11.3 (average 5) and resulting in more diffuse distributions (Fig. 4a).

Smaller apparent step lengths, *l*_*t*_, were also associated with the resting state for all models, and greater values of *l*_*t*_ were associated with the moving state. Across collar deployments, the posterior mean of median apparent step length $\left({e}^{m_i}\right)$ ranged from 7.5 to 20 m (average 13 m) for the resting state and from 59 to 383 m (149) for the moving state. Log standard deviations (*σ*) diverged less between states, with posterior means ranging from 0.8 to 1.0 (average 0.9) for the resting state and 0.8 to 1.7 (average 1.4) for the moving state. This resulted in more diffuse apparent step length distributions for the moving state than the resting state (Fig. 4b). However, apparent step lengths for observations assigned to moving state exhibited negative skewness which was not fully captured by the lognormal distribution (Supplemental Figure 1).

Both *a*_*t*_ and *l*_*t*_ displayed bimodality when aggregated across time. Because this bimodality was apparent for time-aggregated individual time series, we display data aggregated across all 21 collar deployments in Fig. 4c and d. This bimodality and the correlation between *a*_*t*_ and *l*_*t*_ (Fig. 4e) contributed to the model’s ability to separate between states. For example, the most common value of *a*_*t*_ across all 21 collar deployments was the minimum 0.005, which was recorded for 25,411 of 81,024 observations. Of these, only 65 had a *P*(*s*_*t*_ = 1) < 0.95. While we display the time-aggregated data (Fig. 4) to demonstrate the correspondence between observed bimodality and estimated state-dependent probability distribution, it is important to note that this view ignores the time dependence that affects any given observation’s state probability.

The duration of rest events varied within a narrow range across collar deployments and individual fishers. Because we used a time-homogenous model, the duration of resting events is a direct consequence of the probability of remaining in the resting state, *ψ*_1_. The posterior mean of this parameter across collar deployments ranged from 0.89 to 0.96 (average 0.93) for collars set to 15 min increments which corresponds to expected rest event durations of 2.3–6.3 h. For collars set to 30 min increments, the posterior mean of *ψ*_1_ ranged from 0.88 to 0.92 (average 0.91) which corresponds to expected rest event durations of 4.1–6.2 h (Fig. 3). The probability of remaining in the moving state, *ψ*_2_, was more variable across deployments but generally lower than *ψ*_1_ (average posterior mean of 0.88 and 0.79 for 15 and 30 min increment deployments, respectively). As a result, we found that fishers spent more time in the resting state than in the moving state (Table 2) except for summer collar deployments for fishers F01T and F03T. We identified 4068 unique rest events across the 21 collar deployments. The duration of individual rest events was variable, but most lasted between 2 and 8 h (Table 2, Supplemental Figure 2).

**Fig. 5:**
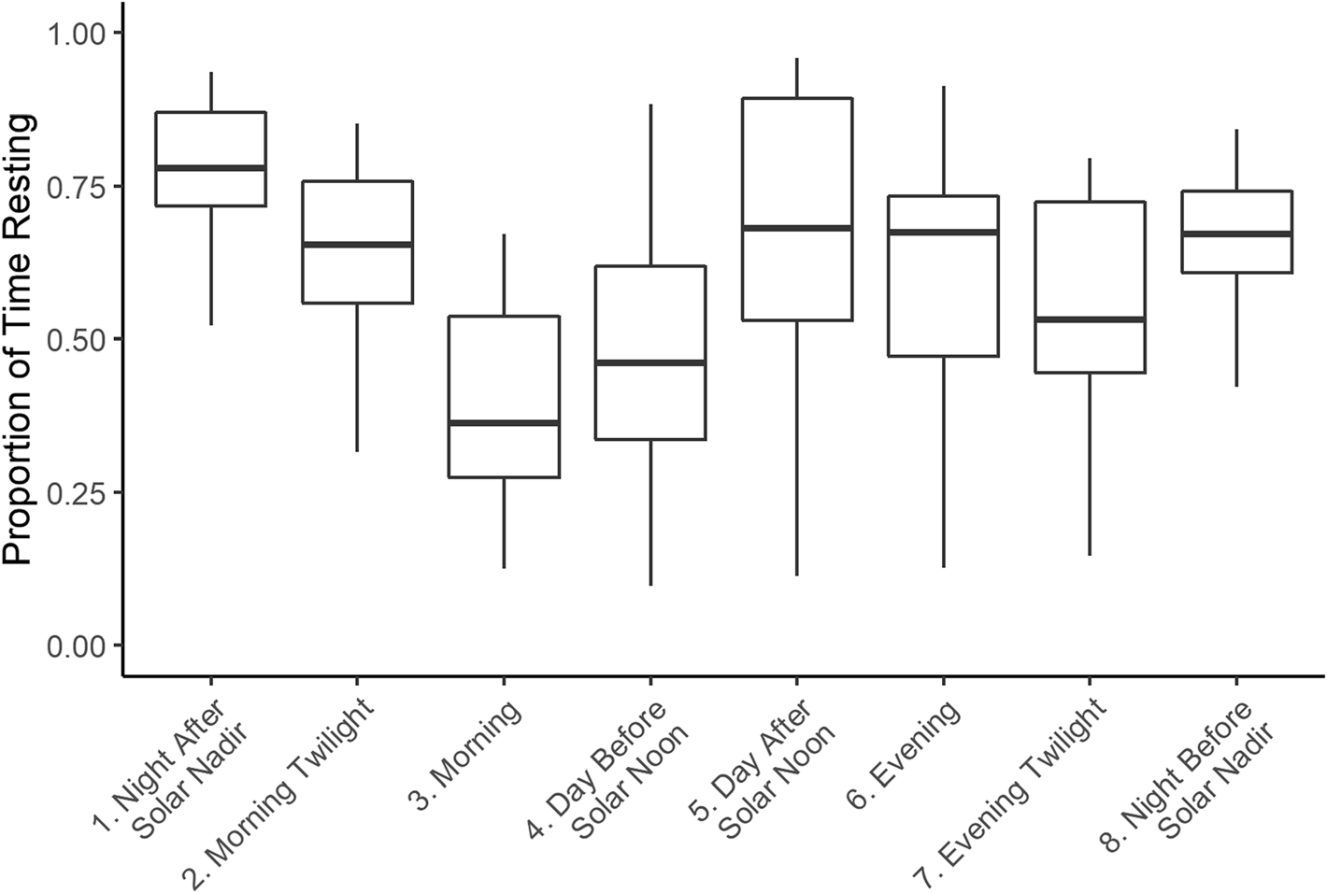
Proportion of time resting by time of day. Categories of daylight are defined as 1) “night after solar nadir” from the solar nadir to the beginning of astronomical twilight; 2) “morning twilight” from the beginning to astronomical twilight to dawn; 3) “morning” from dawn to the end of the golden hour; 4) “day before solar noon” from the end of morning to solar noon; 5) “day after solar noon” was from solar noon to start of the evening golden hour; 6) “evening” from end of day through dusk (evening civil twilight); 7) “evening twilight” from dusk to the end of astronomical twilight; and 8) “night before solar nadir” was from the end of twilight to the solar nadir. In both panels, boxplots summarize over 21 collar deployments depict the median (center line), first and third quartile (lower and upper hinge), with whiskers extending to lesser of the upper/lower range of the data or 1.5 times the interquartile range

The proportion of time resting during each part of the day was variable across fishers and seasons, but in general fishers were least active during the night and most active during the morning (Fig. 5). The proportion of time resting was highest during the night after solar nadir, with a median proportion across collar deployments of 0.78 (range 0.52 to 0.94). For the morning, the median proportion of time resting across collar deployments was 0.36 (range 0.13 to 0.67). The median proportion of time resting during the day after solar noon and during the evening were second and third highest across collar deployments, each at 0.68, but had the highest variability across deployments as reflected in the wide range of proportions (0.11 to 0.95 for day after solar noon, and 0.13 to 0.91 for evening). This may reflect seasonal changes in fisher behavior, as summer deployments of 2016 were on the low end of this range and fall and winter deployments were on the high end. Both night categories had the narrowest range of values in the proportion of time resting (0.42 to 0.84 for night before the solar nadir), which suggests a consistent tendency for our observed fishers to rest at night.

### State space models

**Fig. 6:**
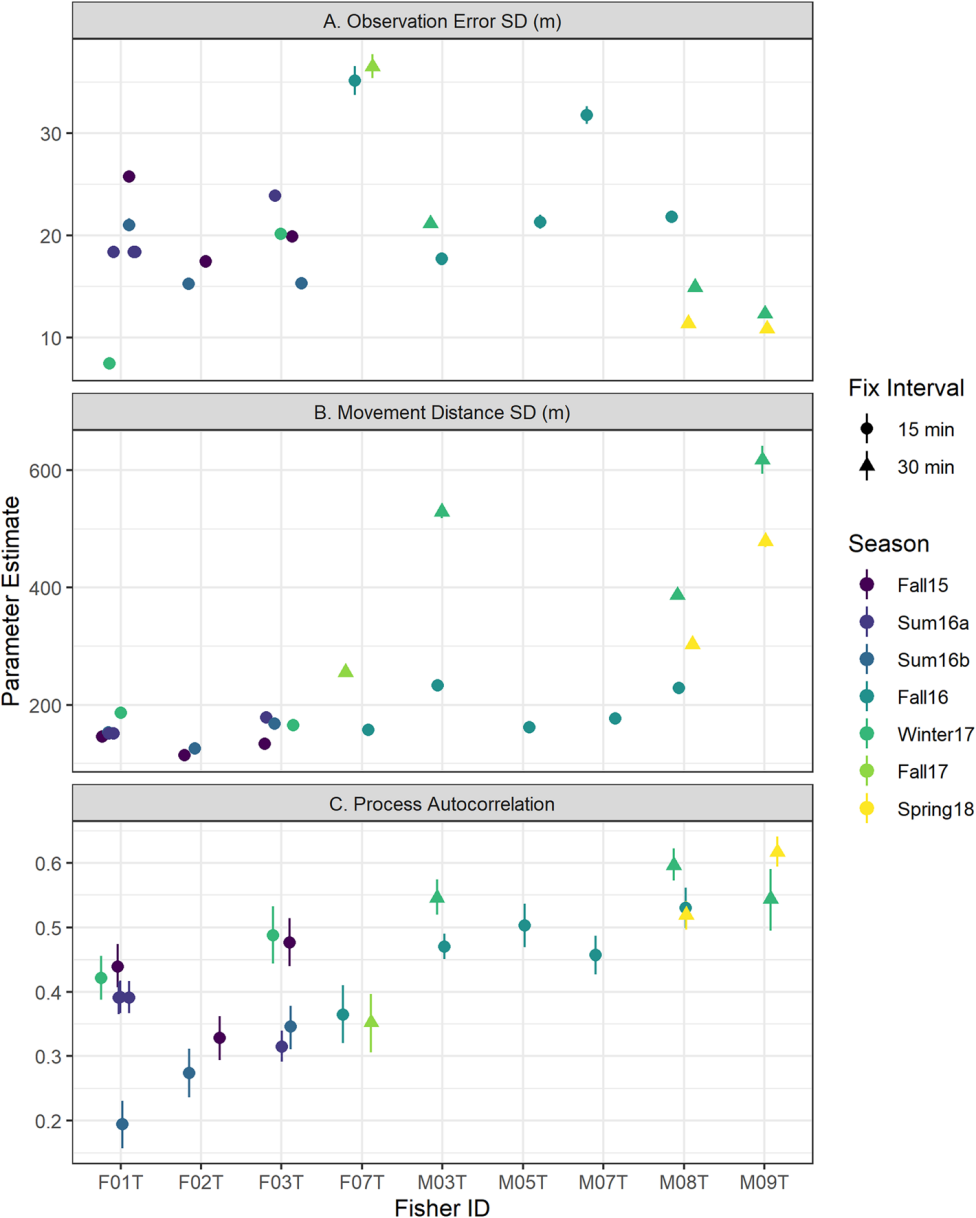
Parameter estimates from state space models (SSMs) fit to state assignments and observed GPS coordinates of 21 collar deployments for fishers. Upper panel depict the observation error standard deviation (*h*) in meters. Middle panel depicts the process autocorrelation (ρ) for the DCRW when the fisher is moving. Bottom panels depict the process error standard deviation (*q*) in meters for when the fisher is moving. All estimates display the posterior mean with error bars representing the 90% uncertainty intervals

As with HMM models, the SSM models fit independently to 21 collars deployment converged successfully to similar estimates. The number of effective posterior samples for the main parameters of the models ranged from 2917 to 4961 (mean 3998) and each parameter had $\hat{R}<1.01$. Parameter estimates displayed broadly similar pattern across all 21 models with observation error a magnitude of order less than process error and positive autocorrelation (Fig. 6).

**Fig. 7:**
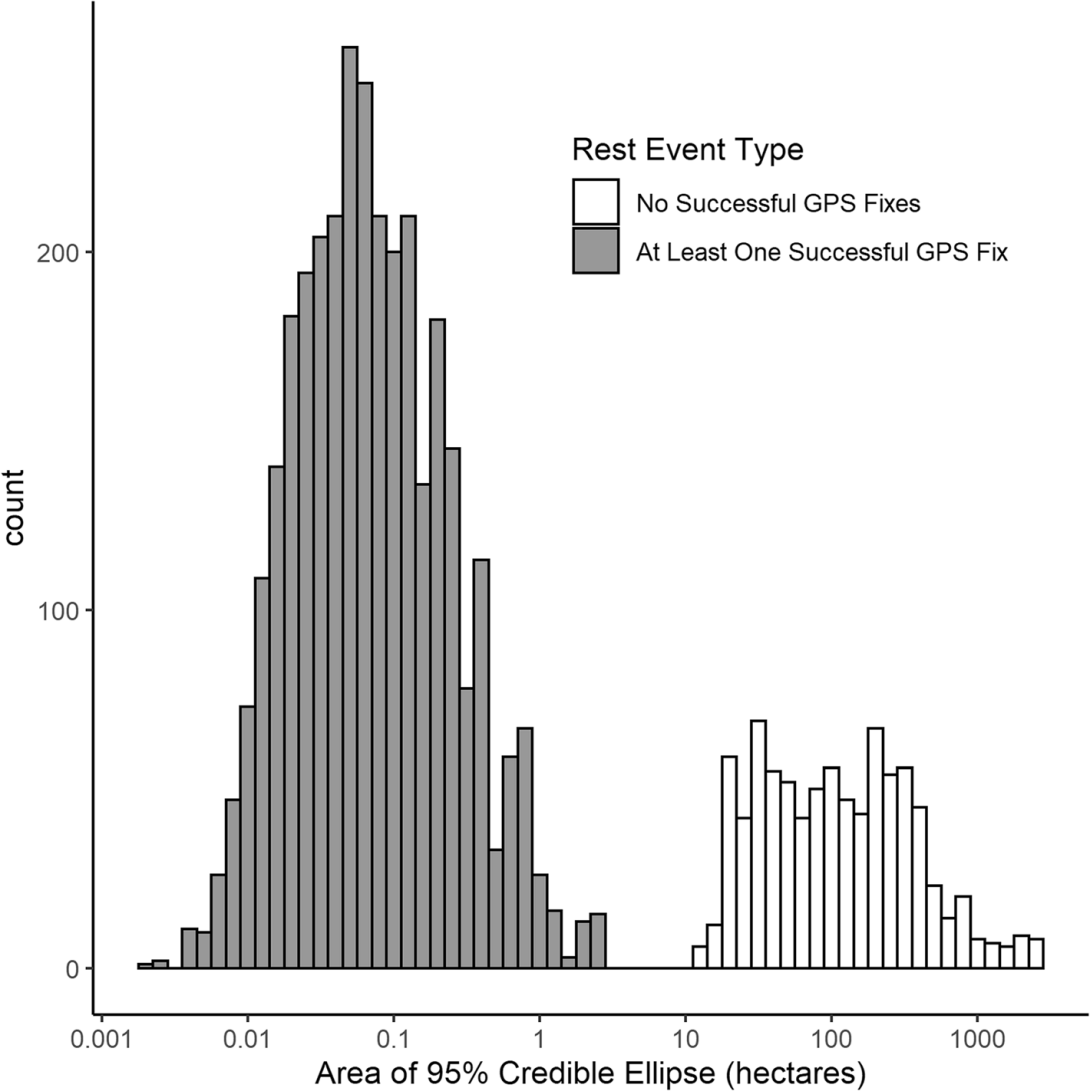
Histogram of the area of 95% credible ellipses for 3772 unique rest events across 21 collar deployments. Note: the x-axis is base-10 logarithm of area

**Fig. 8:**
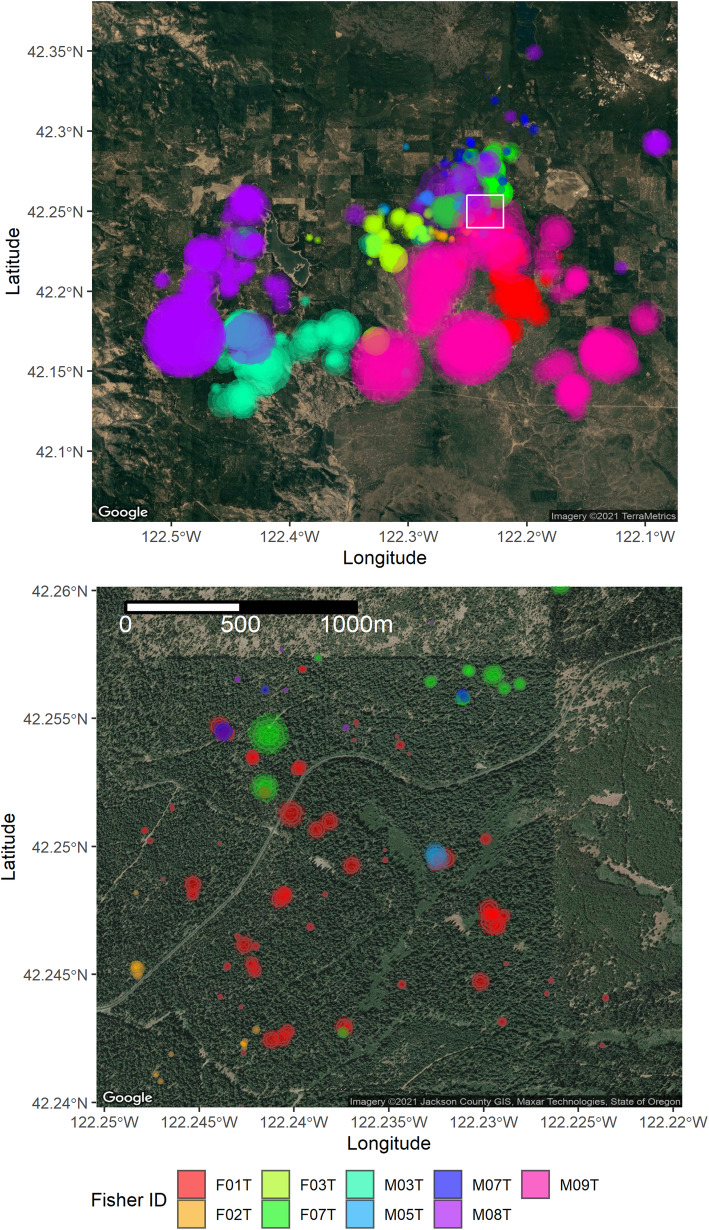
Resting site location estimates over 21 separate collar deployments on nine individual fishers in the study area of the Oregon and California Revested Lands in southern Oregon, USA. The top panel depicts location estimates for all rest events. At the scale of the top panel, only rest events with 95% credible ellipses much greater than 1 ha are apparent. Bottom panel depicts the area within the white box in the top panel and only shows rest events greater than 1 h in duration and with at least one successful GPS fix. Selection in the bottom panel includes two examples of potential reuse of rest sites by multiple fishers. Outer limits of rest event estimates are 95% credible ellipses with two levels of darker shading representing 80 and 50% credible ellipses

The precision of any given rest event location estimate depended on observation error standard deviation and the number of observed GPS fixes. Estimates of observation error standard deviation were on the same order as field tests of GPS precision [56, 57], with an average of posterior means of 20 m (range 7 to 36 m), similar to the average of 21 m estimated precision during field trials [56]. The SSM was able to estimate a location for rest events in the absence of any observed GPS fix with the area 95% credible ellipses for such events (*n* = 850) ranging from 11.8 ha to 2709 ha (Fig. 7). Rest events with the greatest precision were those with at least one successful fix (*n* = 3218) with a median area of 95% credible ellipses of 0.06 ha (range 19 m^2^ to 2.5 ha). While credible ellipses for location estimates of rest events without observed GPS fixes were too large to identify meaningful overlap with other rest events, in all instances, the SSMs constrained the possible location of each fisher within bounds of our study area (Fig. 8, top panel).

We found evidence of reuse of rest sites by individuals fishers over time and by multiple fishers (Fig. 8). Of rest events with at least one successful GPS fix, we identified 1176 geographically distinct areas by spatially joining overlapping 95% credible ellipse. Of these, we found 148 locations were revisited by a fisher after at least 24 h since last use with a median elapsed time between revisits within a single collar deployment of 7.6 days. We found 57 locations where the 95% credible ellipse of the rest event for one fisher overlapped with the 95% credible ellipse for another fisher. Of these, we found two instances of the 95% credible ellipses rest events for three fishers overlapping spatially.

During periods of movement, the scale of fisher movement was an order of magnitude greater than the GPS observation error. The posterior mean standard deviation of process error, which describes variance in the step length between successive fisher locations during periods of movement, averaged 165 m (range 115 m to 233 m) for the 15 collar deployments set at 15 min intervals and 428 m (range 256 m to 618 m) for collar deployments set at 30 min intervals. When fishers were moving, there was a tendency for them to continue moving in the same direction as the previous timestep, as evidenced by the positive correlation in movement direction (*ρ* average posterior mean 0.437).

The simulation smoother of the SSM also estimated the distance moved at each timestep when the fisher was in the moving state. The average posterior mean cumulative distance moved per day (Supplemental Figure 3) ranged from 3.6 km/day (F03T Fall 2015) to 12.8 km/day (M09T Winter 2017) with an overall average of 6.7 km/day (3.5 SD) for females and 7.7 km/day (6.8 SD) for males. The total distance moved per day was interspersed with resting events and so daily movement distances where made up of one or more periods of movement between resting. For these movement events, the average posterior mean cumulative distance travelled between rest events ranged from 0.6 km (F03T Fall15) to 4.2 km (M09T Winter17). For the two female fishers with multiple deployments across seasons (F01T and F03T), we found a similar pattern of greater daily cumulative distance travelled during summer deployments than during fall and winter deployments. This corresponds with a lesser proportion of time resting during summer deployments for these fishers (Table 2), particularly during the day (Fig. 5). However, F02T showed similar distances travelled between fall and summer deployments (Supplemental Figure 3), which suggests that the seasonal patterns observed in F01T and F03T may be due to variation among individuals rather than a general pattern of behavior by female fishers. The greatest daily distance travelled was for fisher M03T, who traversed an estimated 47 km (90% credible interval: 46–48 km) on March 25, 2017. Similar long daily distances were estimated for fisher M09T (35 km on March 11, 2018) and M08T (32 km on March 22, 2017). In contrast, the longest daily distance observed for a female fisher was 17.6 km (90% credible interval: 17.1–18.1 km) for F01T. We suspect that long distance movement of these males reflects mating behavior. During mating season, male fishers may travel to many females during their short period of estrus [58]. The timing of the long-distance movement we observed is consistent with seasonal mating as the average natal den initiation date we observed for this fisher population in a separate study was March 30 [56] and mating takes place approximately 10 days after birth [58].

### Field validation

We identified 79 suspected resting events using VHF telemetry across 21 deployments. Of these 12 (14%) did not correspond temporally with a resting event identified by the HMM models, but for 5 of these 12 the HMM estimated a *P*(*s*_*t*_ = 1) ≥ 0.6. For those 5 events, the location identified via VHF telemetry was within the 95% credible ellipse of the HMM/SSM identified rest event nearest in time to VHF record. Of those which could be matched temporally to an HMM/SSM rest event, 8 (9%) were matched to rest events with no successful GPS fixes and thus large credible ellipses. For all but one of these, the VHF rest location was within the 95% credible ellipse (median area: 25 ha). 22 (25%) of VHF identified rest events were located within the 95% credible ellipse of the temporally corresponding HMM/SSM rest event (median area: 265 m^2^). For another 27 (32%) the VHF identified location was within 25 m (median distance from edge: 6 m) of the nearest edge of the corresponding 95% credible ellipse (median area: 141 m^2^). Finally, 10 (12%) of VHF identified locations were greater than 25 m from the nearest edge (median distance from edge: 52 m) of the corresponding 95% credible ellipse with a maximum distance of 433 m (median). Examples from each of these categories are depicted in the Supplemental Figure 4.

## Discussion

Our two-stage approach to modelling fisher GPS location and accelerometer data allowed us to overcome the dual problems of missing and imperfectly observed location data and unobserved behavioral states to obtain credible estimates of fisher resting sites. The data we analyzed represent the largest published GPS dataset for fishers in North America and allowed us to make inferences across individuals and within individuals across seasons. Despite fitting models independently to each dataset, we found remarkable agreement among the parameter estimates for both the HMMs and SSMs. This suggests that our models captured general patterns of fisher behavior and provided specific estimates of the location of resting sites for these individuals. Thus, we were able to both improve the general state of science on fisher ecology and produce directly actionable information to improve management of the study population.

The models described here allows use of the complete set of collected data to support analysis of fisher use of their landscape. A central tenet of ecology is that animals make movement choices to maximize their fitness [59]. We found that fisher spend a substantial amount of time resting and thus we infer that rest sites are important to their life history and constitute spatially explicit unique habitat components. The use of multiple, well-distributed rest sites suggests that regular access to a rest site is also important. We surmise that rest events have important influence on fisher fitness and therefore the physical location (e.g. slope, aspect), and the surrounding vegetation structure and composition, of the rest structure contribute to the fitness. The precise estimate of rest sites provided by our models gives a complete accounting for likely rest sites from which abiotic and biotic features can be summarized and evaluated. This could lead to a better description of rest site that could facilitate management decisions intended to retain rest site function or create potential rest sites (e.g. selective slash pile retention or creation). The data here should be considered to represent a minimum number and spacing of rest sites because of the relatively short duration of monitoring and the limited number of reused rest sites. We suspect that with a greater monitoring period additional rest sites could be identified.

By using accelerometer-derived activity measurements matched to the sampling interval of GPS positions, we were able to successfully decode latent behavioral states despite GPS measurement error. HMMs and the closely related hidden semi-Markov models [60] applied to location data alone are valid only when measurement error is negligible and GPS position fix rate is high. Measurement error in excess of approximately 10% of the scale of movement can severely affect parameter estimates for movement models [16]. GPS measurement error and low fix rate affects both step lengths and turning angles metrics, but turning angles are more affected [43, 44]. Because accelerometers require only battery power and data storage, they are unaffected by the error associated with satellite and telemetry positioning methods, which rely on transmission of signals between the monitoring device and satellites or telemetry receivers. Accelerometers can collect large amounts of data that can be summarized into multiple statistics appropriate for application in HMMs [13]. The activity measure we used for this analysis was ideal for our purposes because it required only limited battery power and data storage, matched directly to sampling interval of GPS positions resulting in a complete time series with no missing observations, and could be readily modelled with the flexible Beta distribution. While we understood that apparent step lengths were affected by measurement error, the bimodality observed in both metrics and correlation between the two metrics suggest that apparent step lengths provided meaningful information to the HMM likelihood, although, we note that the lognormal distribution did not adequately capture the negative skew of step-lengths for observations assigned to the moving statement. The joint bimodality in the observed data probably contributed to the strong separation in the posterior state assignment probability, where greater than 95% of observations had a state assignment probability in excess of 0.95.

Although the HMMs allowed us to estimate when each fisher was resting, the SSMs allowed us to quantify uncertainty about the location of fisher rest events to achieve our main goal of identifying potential resting sites for the study population. Fishers use forest features such as snags and cavities for resting sites which have been described as forest features of high conservation value [38], but relative importance of such structures is unknown. Camera traps and direct observation through VHF telemetry can confirm use of such features by fishers, but sampling of these sites is usually opportunistic and time consuming. For instance, 9 years of VHF and camera monitoring of 127 fishers led to a similar number of rest occasions (1040) and locations (933) and slightly lower estimates of reuse rates, 9.3% for females and 10.4% for males [61]. Although deducing the number of rest locations was not the goal of either study, our methods provided a similar amount of data to address this question, with a complete resting and movement chronology during the period of observation. Our estimate that 15% of rest locations were reused either by a single fisher or by multiple fishers may imply that these locations may be more important than those used only once. Overlapping, credible ellipses for rest sites suggest potential reuse of specific landscape features that may be more important for fisher ecology than features used only once. However, the overall number of rest locations was much greater than those reused, which suggests that fishers will use many features on the landscape for resting.

Our VHF telemetry field validation showed reasonable agreement with the GPS identified rest events, although far fewer rest events were identified using VHF telemetry. While only about half (43) of the VHF-identified rest locations (85) were inside or within 10 m of the edge of the temporally corresponding rest event 95% credible ellipse, it is not clear that disagreement between the two methods can be attributed to error in one method alone. For example, during our analysis we found a field data entry error on the time of observation that after correcting resulted in a match between the methods. For the two VHF rest events with the greatest distance from the edge of the credible ellipse for temporally corresponding HMM/SSM rest event, the HMM/SSM identified a resting location corresponding to the VHF location but at a different time, but we could not confirm this resulted from a data entry error. On the other hand, we found that the areas of rest site credible ellipses corresponding to VHF rest events which were nearby but not within the credible ellipse tended to be smaller than those for which the VHF rest event was within the ellipse. This could be due to violations of the assumptions of the SSM resulting in location estimates that were too precise. Adjustments to the SSM model, for example by allowing for bias or correlation in GPS measurement error, or non-Gaussian measurement error would have results in more diffuse credible ellipses which would have resulted in greater agreement between the two methods. We propose that direct observation from crew-based telemetry, cameras, and GPS data would best provide an understanding of which structures fishers perceive to be most important for survival and recruitment as the SSMs produced a catalogue of resting event location estimates that can be used to prioritize confirmatory field surveys. Further field validation of the rest sites identified by this study is planned.

Our SSM formulation allowed us to estimate the location of the fisher for each timestep of collar deployment, even when a GPS fix was not acquired. A broad-range of models have been developed to deal with the problem of apparent temporal irregularity in GPS telemetry as a result of missing GPS fixes by parameterizing animal movement as a continuous process that is observed at specific timepoints [62]. For example, continuous time methods have been developed to account for measurement error of observed locations [19], to account for state-switching [63] and have been applied to fishers [27]. Recently, Michelot and Blackwell [64] introduced extend continuous time models to allow for switching between behavioral states between any two irregularly spaced observations. These approaches, particularly that of Michelot and Blackwell [64], are flexible and powerful due to their ability to accommodate correlation across multiple scales and accommodate irregularly observed data. However, because we had an activity measurement and thus a HMM-based estimate of the behavioral state for each scheduled GPS fix, we chose to parameterize the SSMs in discrete time and use the simulation smoother to estimate the location of the fisher for each timestep even if no GPS fix was acquired. This resulted in a complete time-series of both behavioral state and location estimates. The error for time steps with no GPS fix was greater than those with a successful fix, but because we assumed that fishers remained stationary for rest events, rest events with at least one successful fix had markedly greater location estimates than those with none. ‘.

While the relatively simple paired HMMs and SSMs we fit to the data allowed us to achieve our research and management goals, the data structure and results suggest several possible model expansions. We collected location and activity data from nine individual fishers, four females and five males, with collar deployments representing different seasons. These data could be fit to a hierarchical (mixed) model that assumes that individual parameters–HMM state persistence probabilities, HMM beta and lognormal mean and variance terms, or SSM process and observation error standard deviations–arise from common probability distribution across individuals and deployments. Such a model would allow for partial pooling of parameter estimates and quantify variation within and among individuals [65], but at the cost of increased model complexity and computation time. While adopting a hierarchical model allows inference across individuals, it may not improve estimates of state assignment when individual track data are large [53]. Additionally, by modelling all collar deployments in the same model, it would be possible to look for seasonal differences in state persistence. While we did not observe obvious difference in state persistence across seasons, that was not a goal of this study and so we cannot rule out such differences. A second potential avenue for model expansion is inclusion of exogenous variables and temporal heterogeneity HMMs or SSMs. For example, we identified a nocturnal pattern in the proportion of time fishers spent in the resting state. Nocturnal rest structures and microsites were more specific for Pacific martens, dominated by cavities and chambers [66], which were likely more beneficial for thermoregulation than similar structures used during the day. Nonetheless, finding such structures with human observers is nearly exclusively completed during the day. Understanding influence and timing of fishers’ preferential resting periods could be more directly modelled by allowing state persistence probabilities in the HMMs to vary based on time of day [8]. Additionally, the observed lack of fit of apparent steps lengths to the lognormal distribution in the moving state may be indicative of an additional behavioral state or of temporal heterogeneity in movement that could be better captured by a three-state and/or time-inhomogeneous HMM. Because we were not interested in inferring anything about step lengths in the HMM step due to the confounding of measurement error and because our focus on this analysis was on identifying resting sites, we were less concerned about this lack of fit than if our inferential goal was on characterizing fisher movement behavior. For the SSMs, one area of expansion is to allow measurement error to vary based on recorded GPS quality measurements, such as horizontal dilution of precision. Similarly, we could explore whether fishers move greater distances during the day by allowing process error to vary based on time of day or weather. Conceivably, spatially-varying variables (e.g., local forest structure, distance from roads) can also affect fisher movement and behavior. However, including these variables is more complicated because of GPS measurement error and missing fixes. One way to address this problem involves discretizing the continuous spatial state into a spatial grid [18]. However, grid cell size would need to be carefully chosen to ensure that spatial covariates (e.g., forest stand type and structure) are approximately homogenous within a cell and are at scales meaningful to forest management and fisher ecology and to ensure reasonable computation time. Finally, reuse of rest sites suggests an alternative HMM parameterization involving “centers of attraction” where fishers may switch between a moving state and a “resting” state centered around a particular resting site [67]. This approach has the advantage of estimating the location of only a single center of attraction for each reused rest site, as opposed to our approach of identifying reuse that relied on the overlap of unconstrained location estimates. However, because the number of centers of attraction would need to be specified a priori in such a model, our approach remains useful as a first step to inform the construction of such a model. Further, fishers moved an average of 7.4 km daily, suggesting the capacity to move among most rest structures within a short period. If their territory boundaries are patrolled regularly, similar to every 4.6 days on average for Pacific martens (*Martes caurina*) [68], then territorial boundaries may also be coded as a movement attraction boundary.

## Conclusions

Here, we demonstrated that a relatively simple two-stage approach can overcome the twin challenges of latent behavioral states and measurement error in the context of management of sensitive species. By leveraging accelerometer data to resolve latent states in a set of HMMs, we were able to use HMM output in a set of SSMs to quantify location error to produce a set of rest event location estimates. The models provided both basic information on fisher ecology such as rest event duration, diurnal patterns of resting, cumulative distance moved between rest events, and specific information on locations of rest events that can be used to inform conservation decisions. Our relatively simple method produced rapid results and can be readily applied to additional collar deployments. Given the flexibility of both HMMs and SSMs, either stage of the model can also be expanded to include hierarchical structure or appropriate covariates for additional ecological insights.

## Supplementary Information

**Additional file 1: Supplemental Figure 1.** Comparison of observed activity (left two panels) and observed apparent step-lengths (right-panel) to estimated state-dependent beta and lognormal probability distribution, respectively. The probability distributions lines consist of 100 independent draws from the joint. **Supplemental Figure 2.** Duration of rest events (hours) for 21 GPS collar deployments on 9 individual fishers. A rest event is defined as continuous segments of time series for which *P*(*s*_*t*_ = 1) > 0.95. **Supplemental Figure 3.** Cumulative distance moved per day for 21 GPS collar deployments of 9 individual fishers. Points depict the posterior mean cumulative distance moved per day. Error bars on the points depict the 5^th^ and 95^th^ percentile of the posterior distribution. Boxplots are based off the posterior mean cumulative distance moved between each rest event and depict median (center line), first and third quartile (lower and upper hinge), with whiskers extending to 5^th^ and 95^th^ percentile of the posterior mean. **Supplemental Figure 4a.** Example comparisons from field validation of HMM/SSM identified rest events and contemporaneous radio telemetry identified rest events. Of 67 suspected rest events radio telemetry resting locations which could be matched in time to a HMM/SSM identified rest events, 8 were matched to rest events with no successful GPS fixes and large credible ellipses. Four of these are depicted if Figure 4a including the only example where the radio telemetry rest event was outside of the 95% credible ellipse for the HMM/SSM resting location (bottom right panel). The yellow target symbol is the radio telemetry identified resting location, and the white contours represent the 50%, 80%, and 95% credible ellipses for the HMM/SSM resting location. **Supplemental Figure 4b.** Example comparisons from field validation of HMM/SSM identified rest events and contemporaneous radio telemetry identified rest events. Of 67 suspected rest events radio telemetry resting locations which could be matched in time to a HMM/SSM identified rest events, 22 lay within the 95% credible ellipse estimated from the HMM/SSM. Four of these are depicted in Figure 4b. The yellow target symbol is the radio telemetry identified resting location, the red points are all GPS location fixes associated with the rest event, and the white contours represent the 50%, 80%, and 95% credible ellipses for the HMM/SSM resting location. **Supplemental Figure 4c.** Example comparisons from field validation of HMM/SSM identified rest events and contemporaneous radio telemetry identified rest events. Of 67 suspected rest events radio telemetry resting locations which could be matched in time to a HMM/SSM identified rest events, 27 were outside of the 95% credible ellipse but within 25m of the nearest edge. Four of these are depicted in Figure 4c. The yellow target symbol is the radio telemetry identified resting location, the red points are all GPS location fixes associated with the rest event, and the white contours represent the 50%, 80%, and 95% credible ellipses for the HMM/SSM resting location. **Supplemental Figure 4d.** Example comparisons from field validation of HMM/SSM identified rest events and contemporaneous radio telemetry identified rest events. Of 67 suspected rest events radio telemetry resting locations which could be matched in time to a HMM/SSM identified rest events, 10 were located further then 25 meters from the nearest edge of the corresponding HMM/SSM 95% credible ellipse. Four of these are depicted in Figure 4d including the example furthest from the closet edge where the radio telemetry rest event was identified 433 meters away from the 95% credible ellipse for the HMM/SSM resting location (bottom right panel). The yellow target symbol is the radio telemetry identified resting location, and the white contours represent the 50%, 80%, and 95% credible ellipses for the HMM/SSM resting location.

**Additional file 2:** Complete R and Stan code for reproducing the statistical analyses in this manuscript.

## Data Availability

The dataset supporting conclusion in this article is included with the article and its additional file. Complete R and Stan code for reproducing the statistical analyses in this manuscript is provided in Additional file 2. Location data have been centered to remove reference to physical locations, but original data is available via email.

## Abbreviations

HMM: Hidden Markov model
SSM: State space model
DCRW: First difference correlated random walk
FFBS: Forward-filtering, backward-sampling
KF: Kalman filter
GPS: Global Positioning System
O&C Lands: Oregon and California Railroad Revested Lands
HMC: Hamiltonian Monte Carlo
BLM: Bureau of Land Management
