# Correction to: Identifying resting locations of a small elusive forest carnivore using a two-stage model accounting for GPS measurement error and hidden behavioral states

**DOI:** 10.1186/s40462-021-00262-w

**Published:** 2021-04-23

**Authors:** Dalton J. Hance, Katie M. Moriarty, Bruce A. Hollen, Russell W. Perry

**Affiliations:** 1 US Geological Survey, Western Fisheries Research Center, Columbia River Research Laboratory, Cook, WA 98605 USA; 2 National Council for Air and Stream Improvement, Inc., Corvallis, OR USA; 3 USDI Bureau of Land Management State Office, Portland, OR USA

**Correction to: Mov Ecol 9, 17 (2021)**

**https://doi.org/10.1186/s40462-021-00256-8**

Following publication of the original article [1], the authors flagged that due to a typesetting mistake, the Additional file 2 (a zip file containing the data and code necessary to replicate the analysis) was omitted from the article.

Additional file 2 is attached to this Correction and has been added to the original article. The publisher apologises to the authors and readers for the inconvenience caused by this omission.

## Supplementary Information

**Additional file 2.** Complete R and Stan code for reproducing the statistical analyses in this manuscript.
