# Supplementary material for: Identifying resting locations of a small elusive forest carnivore using a two-stage model accounting for GPS measurement error and hidden behavioral states
Source: Mov Ecol. 2021 Apr 6;9:17. doi: 10.1186/s40462-021-00256-8 (PMC8025504; doi:10.1186/s40462-021-00256-8)

**Supplemental Materials for “Identifying resting locations of a small elusive forest carnivore using a two-stage model accounting for GPS measurement error and hidden behavioral states”**

**Supplemental Figure 1:**  Comparison of observed activity (left two panels) and observed apparent step-lengths (right-panel) to estimated state-dependent beta and lognormal probability distribution, respectively. The probability distributions lines consist of 100 independent draws from the joint posterior for each collar deployment.
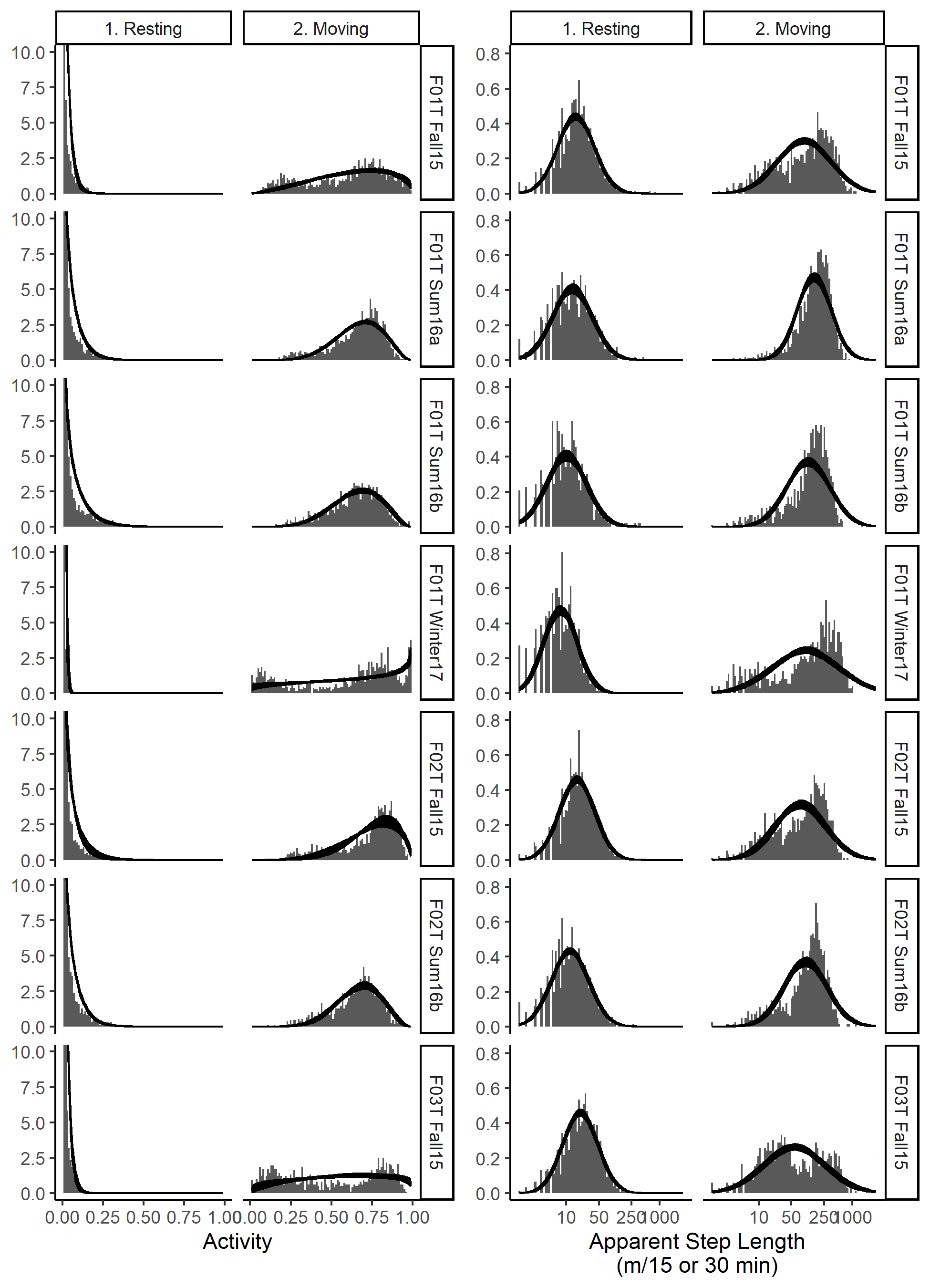


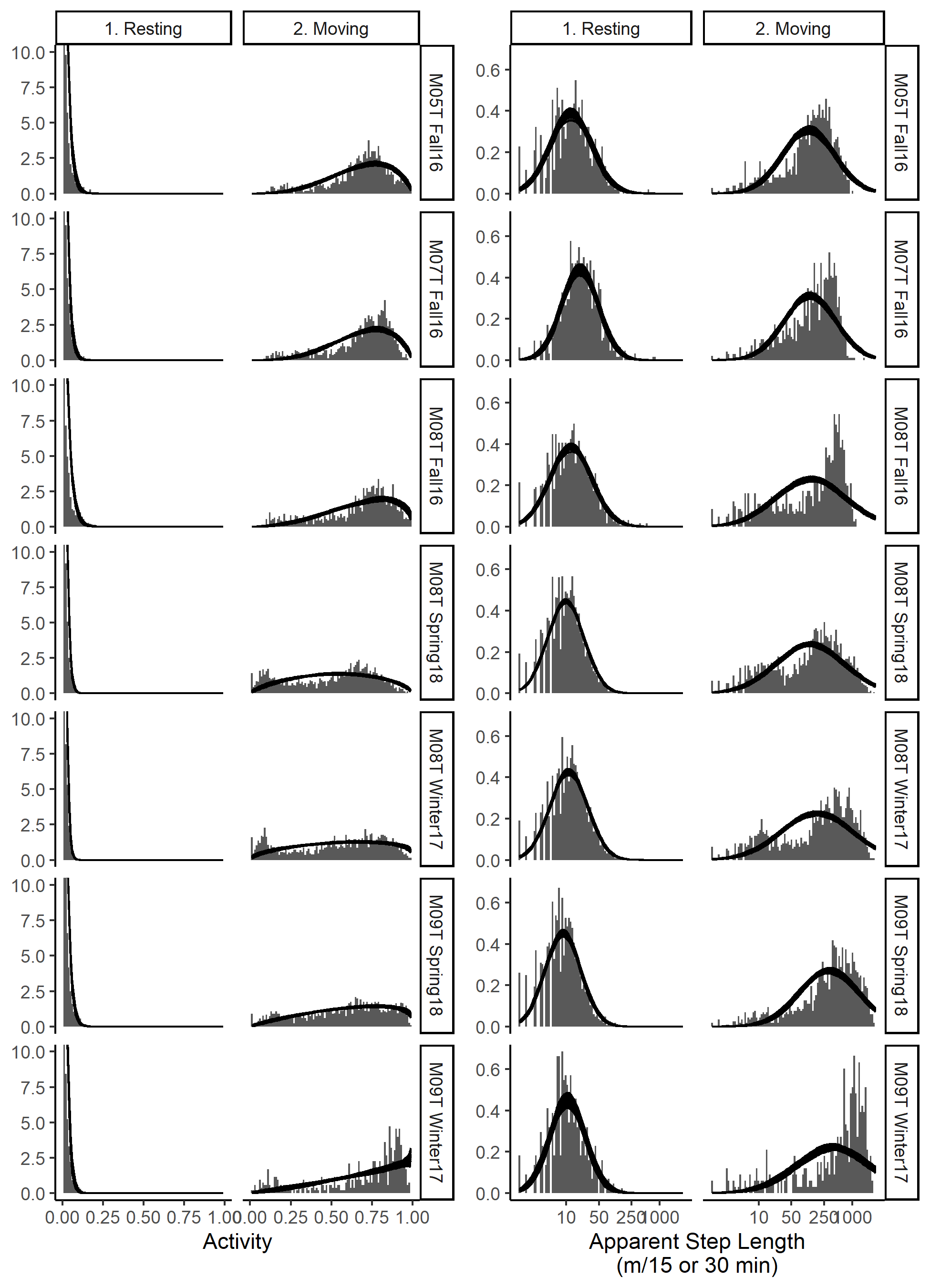

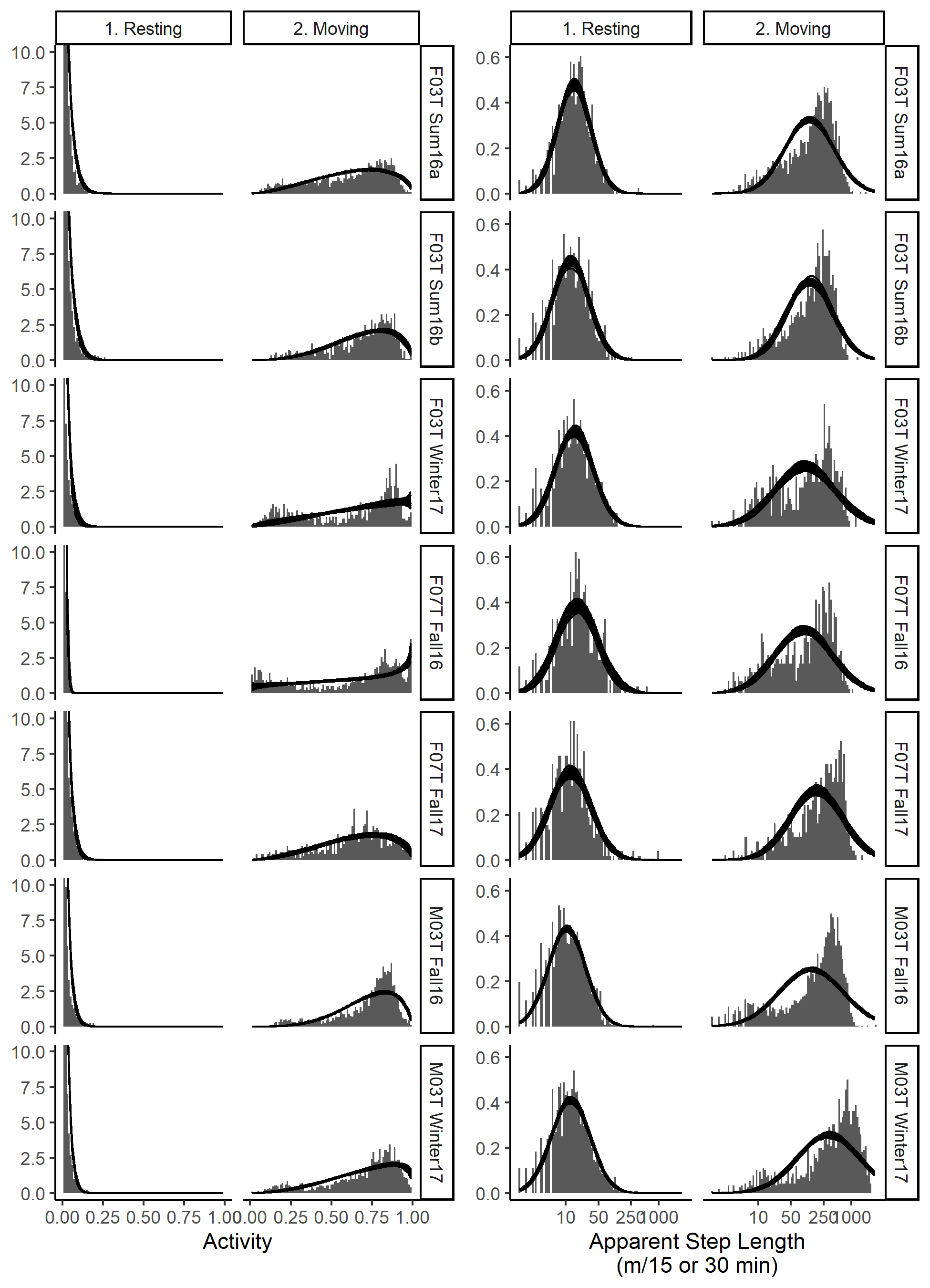


**Supplemental Figure 2:**  Duration of rest events (hours) for 21 GPS collar deployments on 9 individual fishers. A rest event is defined as continuous segments of time series for which $P\left( s_{t}=1 \right)$ > 0.95.


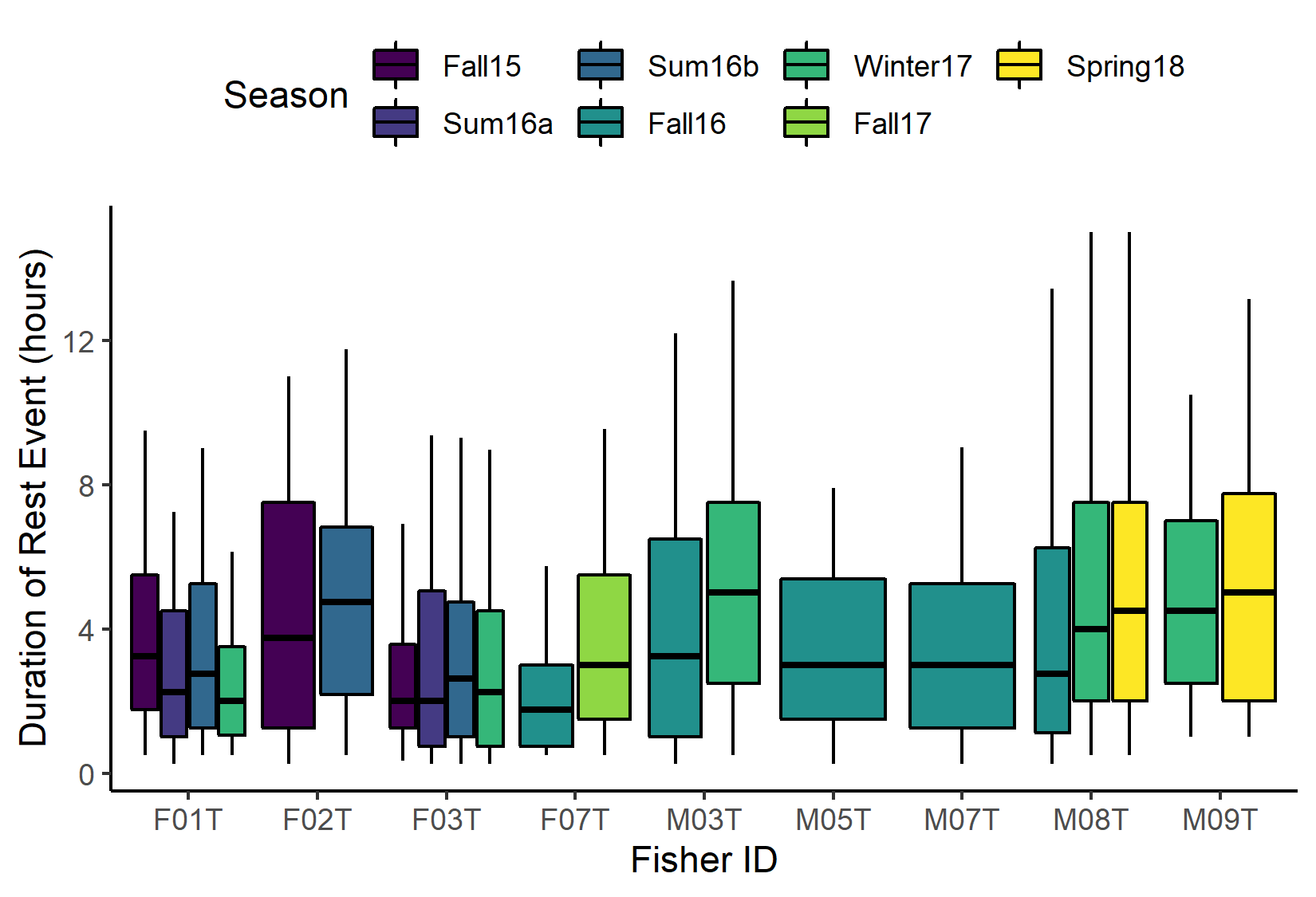


**Supplemental Figure 3:**  Cumulative distance moved per day for 21 GPS collar deployments of 9 individual fishers. Points depict the posterior mean cumulative distance moved per day. Error bars on the points depict the 5^th^ and 95^th^ percentile of the posterior distribution. Boxplots are based off the posterior mean cumulative distance moved between each rest event and depict median (center line), first and third quartile (lower and upper hinge), with whiskers extending to 5^th^ and 95^th^ percentile of the posterior mean.


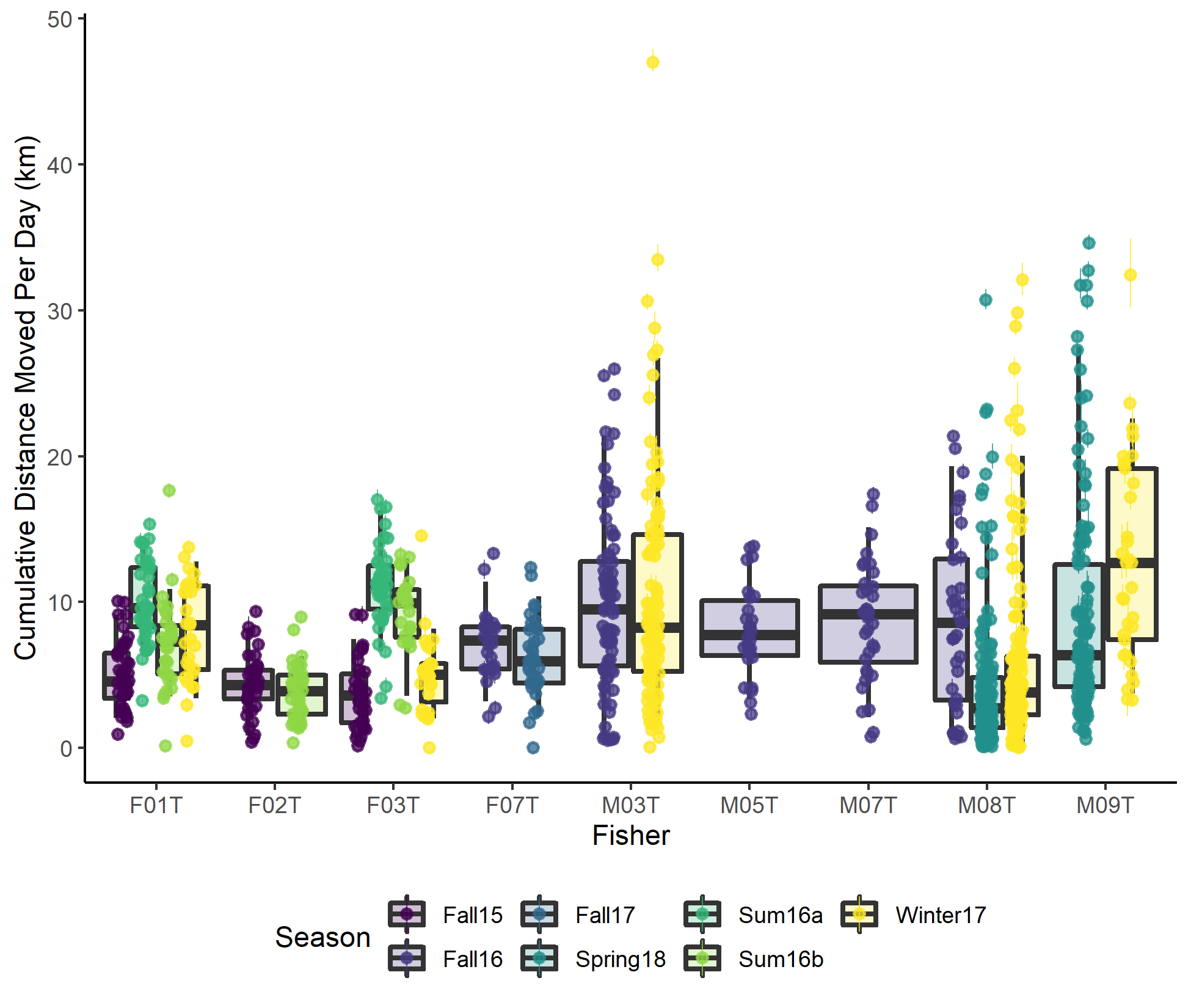


**Supplemental Figure 4a:** Example comparisons from field validation of HMM/SSM identified rest events and contemporaneous radio telemetry identified rest events. Of 67 suspected rest events radio telemetry resting locations which could be matched in time to a HMM/SSM identified rest events, 8 were matched to rest events with no successful GPS fixes and large credible ellipses. Four of these are depicted if Figure 4a including the only example where the radio telemetry rest event was outside of the 95% credible ellipse for the HMM/SSM resting location (bottom right panel). The yellow target symbol is the radio telemetry identified resting location, and the white contours represent the 50%, 80%, and 95% credible ellipses for the HMM/SSM resting location.


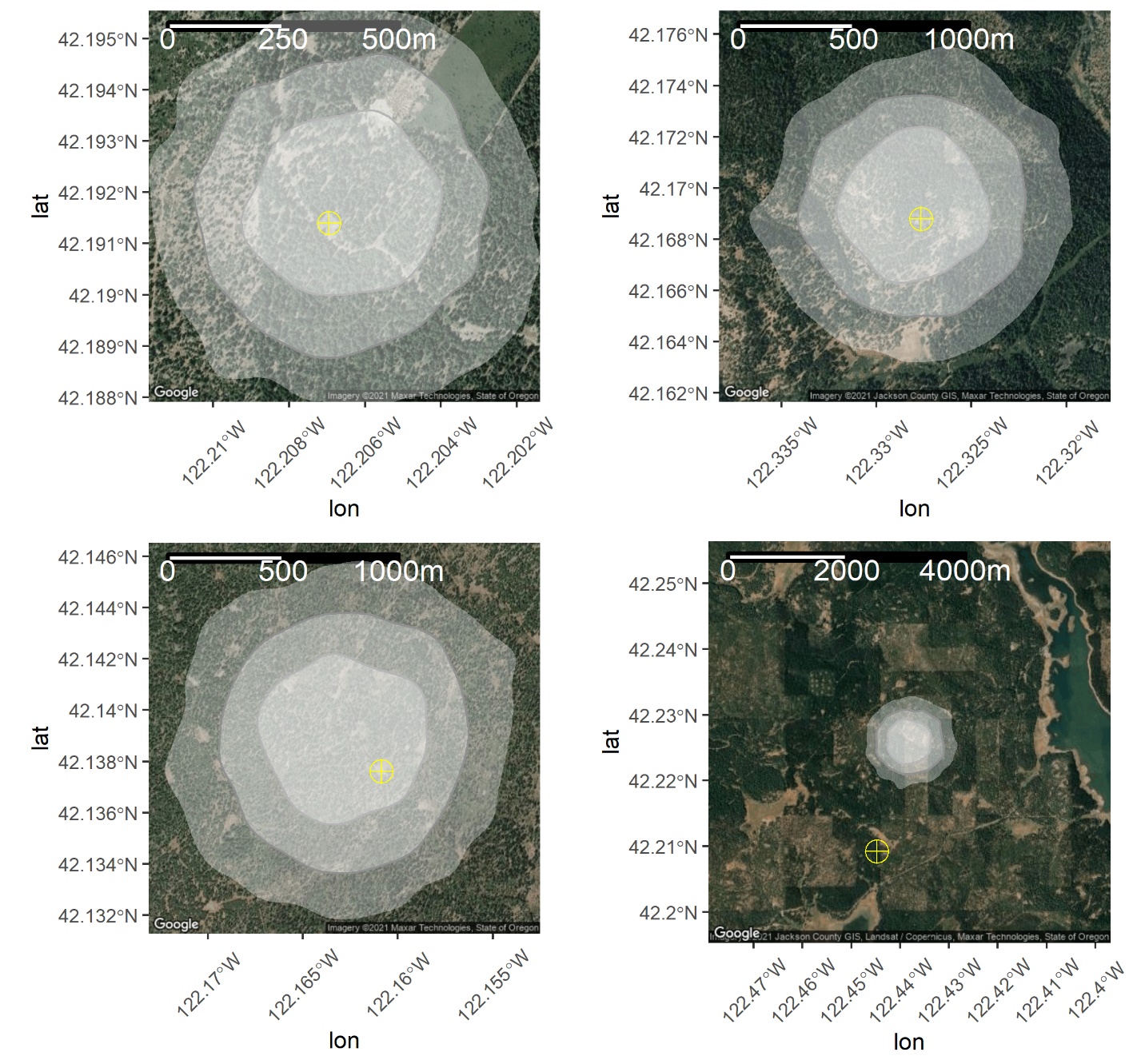


**Supplemental Figure 4b:** Example comparisons from field validation of HMM/SSM identified rest events and contemporaneous radio telemetry identified rest events. Of 67 suspected rest events radio telemetry resting locations which could be matched in time to a HMM/SSM identified rest events, 22 lay within the 95% credible ellipse estimated from the HMM/SSM. Four of these are depicted in Figure 4b. The yellow target symbol is the radio telemetry identified resting location, the red points are all GPS location fixes associated with the rest event, and the white contours represent the 50%, 80%, and 95% credible ellipses for the HMM/SSM resting location.


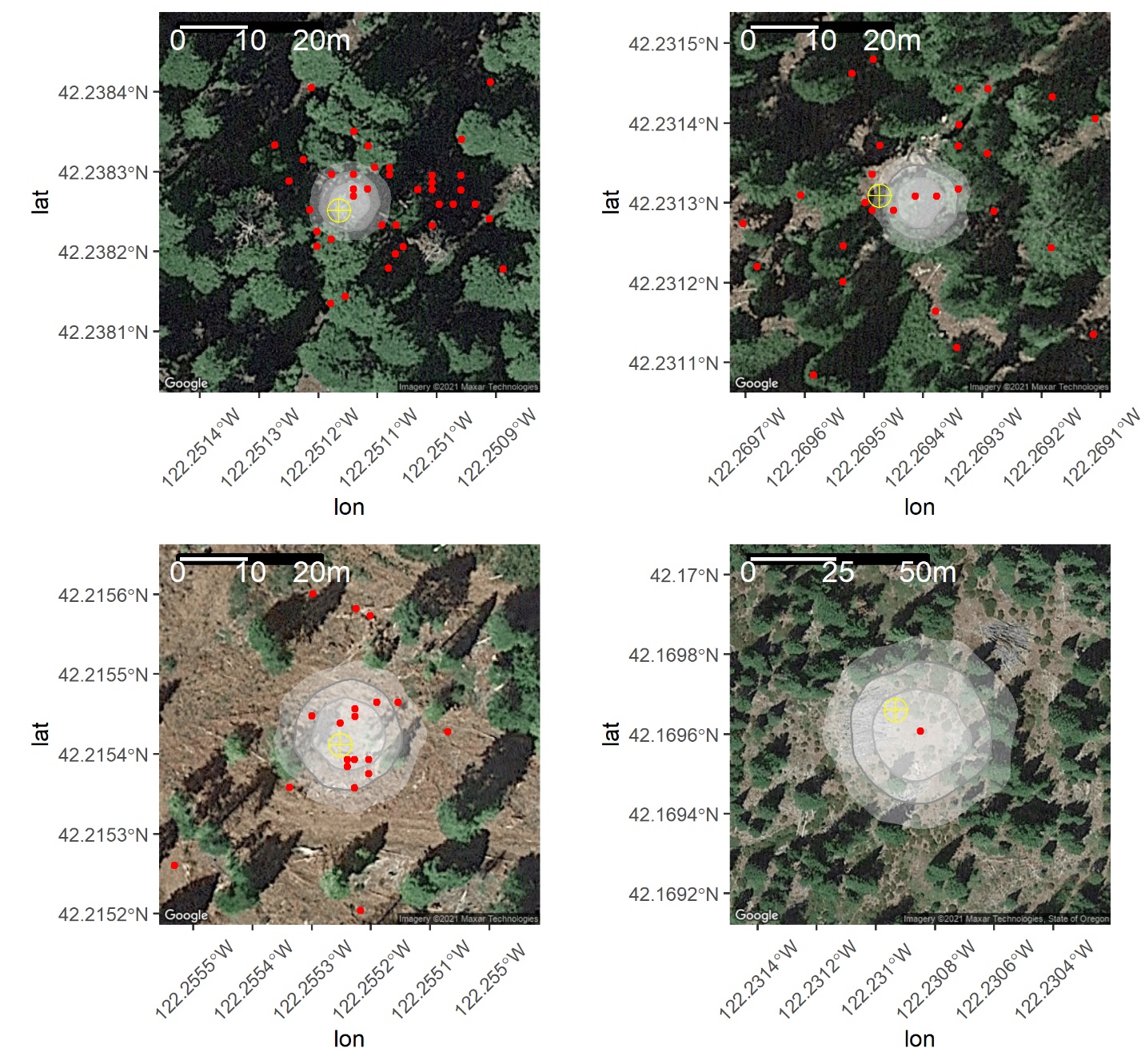


**Supplemental Figure 4c:** Example comparisons from field validation of HMM/SSM identified rest events and contemporaneous radio telemetry identified rest events. Of 67 suspected rest events radio telemetry resting locations which could be matched in time to a HMM/SSM identified rest events, 27 were outside of the 95% credible ellipse but within 25m of the nearest edge. Four of these are depicted in Figure 4c. The yellow target symbol is the radio telemetry identified resting location, the red points are all GPS location fixes associated with the rest event, and the white contours represent the 50%, 80%, and 95% credible ellipses for the HMM/SSM resting location.


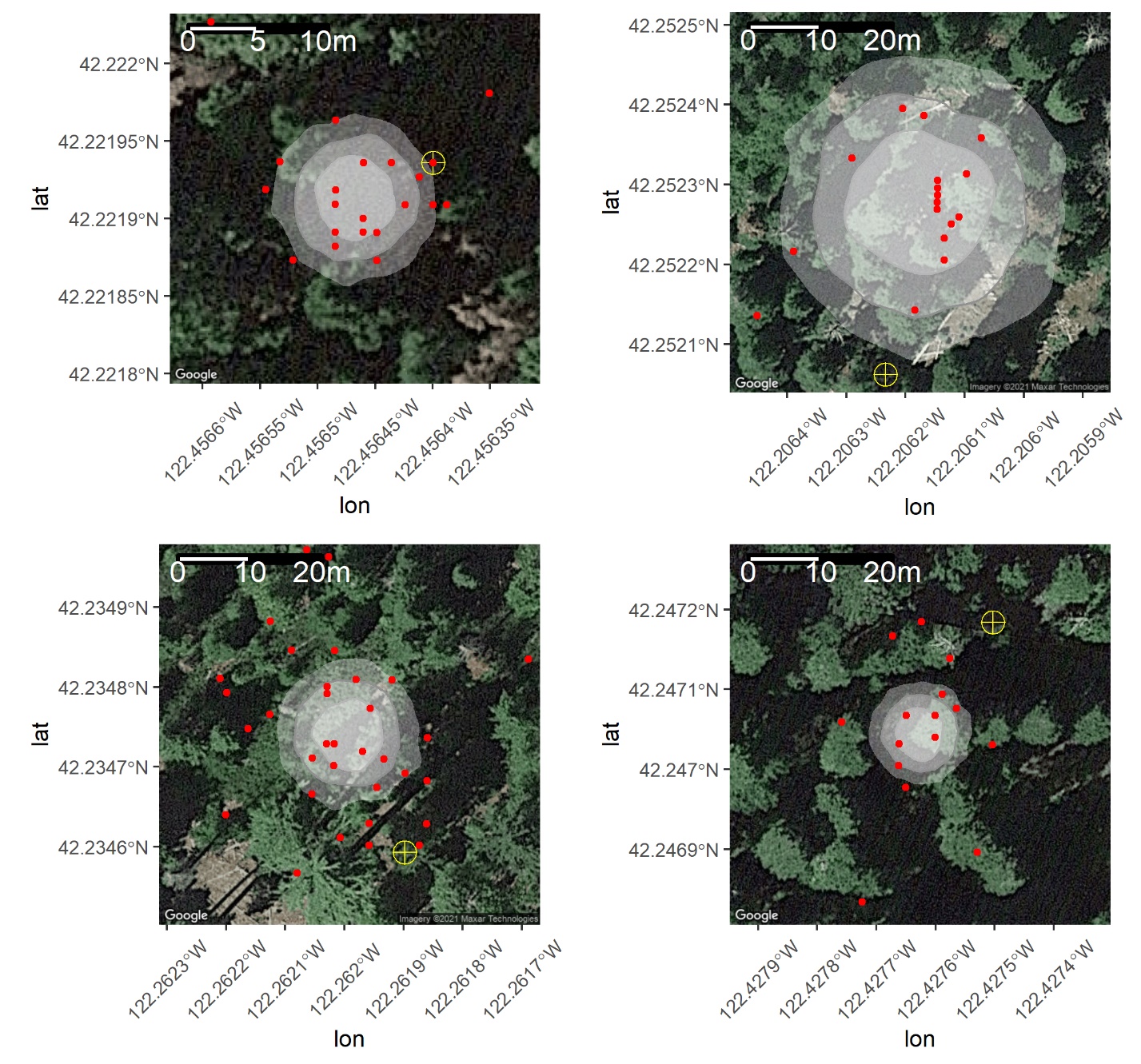


**Supplemental Figure 4d:** Example comparisons from field validation of HMM/SSM identified rest events and contemporaneous radio telemetry identified rest events. Of 67 suspected rest events radio telemetry resting locations which could be matched in time to a HMM/SSM identified rest events, 10 were located further then 25 meters from the nearest edge of the corresponding HMM/SSM 95% credible ellipse. Four of these are depicted in Figure 4d including the example furthest from the closet edge where the radio telemetry rest event was identified 433 meters away from the 95% credible ellipse for the HMM/SSM resting location (bottom right panel). The yellow target symbol is the radio telemetry identified resting location, and the white contours represent the 50%, 80%, and 95% credible ellipses for the HMM/SSM resting location.


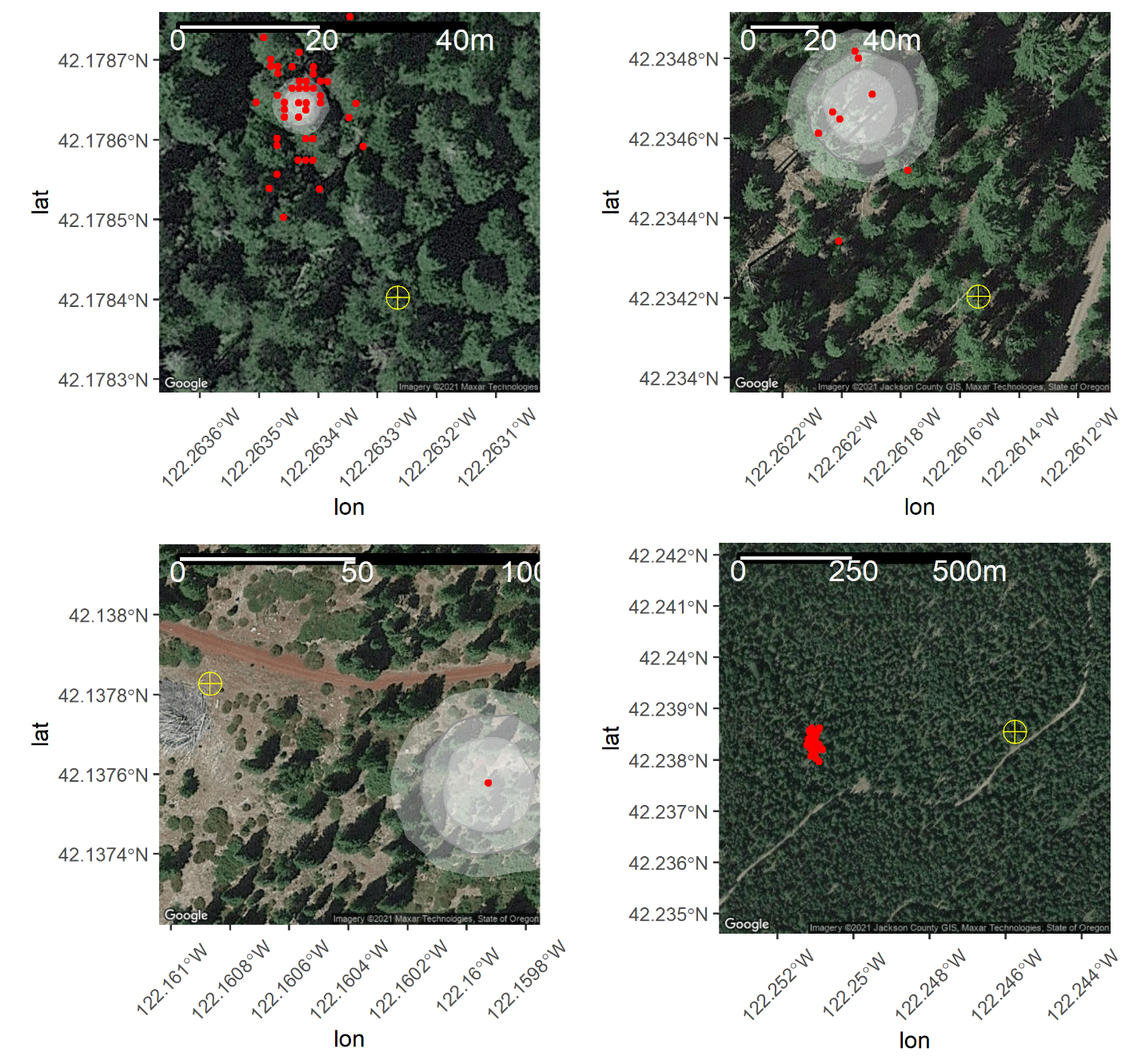

Supplement: Supplementary file 1 — Additional file 1: Supplemental Figure 1. Comparison of observed activity (left two panels) and observed apparent step-lengths (right-panel) to estimated state-dependent beta and lognormal probability distribution, respectively. The probability distributions lines consist of 100 independent draws from the joint. Supplemental Figure 2. Duration of rest events (hours) for 21 GPS collar deployments on 9 individual fishers. A rest event is defined as continuous segments of time series for which P(st = 1) > 0.95. Supplemental Figure 3. Cumulative distance moved per day for 21 GPS collar deployments of 9 individual fishers. Points depict the posterior mean cumulative distance moved per day. Error bars on the points depict the 5th and 95th percentile of the posterior distribution. Boxplots are based off the posterior mean cumulative distance moved between each rest event and depict median (center line), first and third quartile (lower and upper hinge), with whiskers extending to 5th and 95th percentile of the posterior mean. Supplemental Figure 4a. Example comparisons from field validation of HMM/SSM identified rest events and contemporaneous radio telemetry identified rest events. Of 67 suspected rest events radio telemetry resting locations which could be matched in time to a HMM/SSM identified rest events, 8 were matched to rest events with no successful GPS fixes and large credible ellipses. Four of these are depicted if Figure 4a including the only example where the radio telemetry rest event was outside of the 95% credible ellipse for the HMM/SSM resting location (bottom right panel). The yellow target symbol is the radio telemetry identified resting location, and the white contours represent the 50%, 80%, and 95% credible ellipses for the HMM/SSM resting location. Supplemental Figure 4b. Example comparisons from field validation of HMM/SSM identified rest events and contemporaneous radio telemetry identified rest events. Of 67 suspected rest events radio telemetry r [file 40462_2021_256_MOESM1_ESM.docx]
